# Estimating the effect of familiarity in surgical teams on procedure time under a causal graphical model

**DOI:** 10.1007/s10729-026-09780-8

**Published:** 2026-10-30

**Authors:** Gulin Tuzcuoglu, Cagla Keceli, Amber Kratochvil, Kiran Turaga, Daniel Adelman

**Affiliations:** 1 The University of Chicago Booth School of Business, Chicago, IL 60637 USA; 2 The University of Chicago Pritzker School of Medicine, Chicago, IL 60637 USA; 3 The Yale School of Medicine, New Haven, CT 06510 USA

**Keywords:** Team familiarity, Operating room efficiency, Hospital operations, Structural causal modeling

## Abstract

This study estimates the causal impact of surgical team familiarity on surgery duration across various procedures in a hospital, under an assumed Directed Acyclic Graph (DAG) structure. While existing literature indicates that greater familiarity among surgical team members, measured by their shared work experience, is linked to reduced surgery times, these studies typically focus on a specific type of surgical procedure and only suggest a correlational relationship. Our study is the first to examine the hospital-wide relationship between surgical team familiarity and procedure duration using a formal causal inference framework. To explore this relationship, team familiarity is measured based on interaction frequency, and data across procedures are standardized to facilitate analysis via structural causal modeling and regression analysis. An analysis of 33,374 procedures performed in a high-volume academic medical center in the US from January 2017 to November 2023 reveals a negative causal relationship between surgeon-scrub familiarity and surgery duration under our DAG assumptions. Specifically, increasing familiarity by one standard deviation leads to a reduction of 1.9 to 3.7 minutes in surgery duration. Similar, albeit smaller, effects are observed for surgeon-circulator familiarity, whereas no significant impact is found for scrub-circulator familiarity. To our knowledge, this is the first study to estimate the causal effect of surgical team familiarity across an entire hospital’s surgical portfolio under the assumptions of a DAG, moving beyond prior correlational analyses limited to specific specialties or methods. Ultimately, the study reveals that improving team familiarity, particularly between surgeons and scrub nurses, could hold significant implications for improving surgical team efficiency. This is especially relevant in high-volume hospitals, where even minor time savings can accumulate significantly, underscoring the value of leveraging team familiarity to optimize performance in hospital operating rooms.

## Highlights

 

- We explore a potential causal link between surgical team familiarity and procedure duration across various surgical specialties within a hospital.
- We utilize historical observational data and structural causal modeling (SCM) with directed acyclic graphs (DAGs) to analyze the implied causal relationships. We employ ordinary least squares (OLS) regression to estimate the causal effects.
- We find statistically significant negative coefficients for surgeon-scrub familiarity across all models tested, indicating reduced surgery duration with increased familiarity. Surgeon-circulator familiarity also shows negative coefficients, though smaller in effect size.
- We emphasize the importance of enhancing team familiarity, particularly between surgeons and scrub nurses, to optimize surgical team efficiency, especially in high-volume hospitals where minor time savings can accumulate.

## Introduction

Many workplaces rely on temporary teams that convene to accomplish complex tasks. One of the workplaces where teamwork plays a critical role is the operating room (OR). A surgical team in the OR consists of a surgeon, an anesthesiologist, scrub and circulator nurses at its core. While there is a multitude of factors affecting the outcome and duration of surgery, ineffective team communication is among the most frequently cited problems in the OR [18, 23] and is a major cause of errors in the OR [34] and hospitals [56]. The essential aspects of effective teamwork encompass individual and collective expertise, including technical and decision-making skills, adaptability, and interpersonal abilities. Collaborative experiences contribute to the formation of shared mental models of team cognition, ultimately improving efficiency. A prior study found a 0.38 correlation between team cognition and overall team performance [9]. In line with the research establishing the importance of surgical teaming, the Institute of Medicine has advocated for prioritizing effective teamwork in patient care across hospital systems [40]. Thus, it is crucial to understand the effect of surgical team familiarity, i.e. the amount of shared work experience among surgical team members, to enhance teamwork.

In this paper, our main focus revolves around understanding how teaming influences the amount of time a surgical procedure takes. Specifically, we zoom in on the level of familiarity between the surgeon and two key team members: the scrub nurse and the circulator nurse. While anesthesiologists have important responsibilities at the start and end of surgeries, their role typically doesn’t require extensive collaboration with other team members. Thus, they are excluded from our analysis as their assignments are typically ad-hoc and not influenced by systematic factors under investigation. In contrast, the surgeon and the scrub nurse work closely together within the sterile surgical field^1^, and the circulating nurse, although in the nonsterile area, also actively collaborates with the surgeon.

The primary outcome measure in this study is procedure time, which serves as a direct and robust indicator of team efficiency and coordination. Unlike more distal outcomes such as mortality or complications, which are influenced by numerous confounding factors and are often rare events, procedure time directly reflects the impact of intraoperative team familiarity. Its use as a key outcome in assessing surgical team performance is well-supported by existing literature, and the observed efficiency gains have significant managerial relevance for optimizing operating room utilization and overall surgical capacity [4, 6, 14, 16, 31].

In typical hospital environments, a figure such as a nurse manager often non-randomly assigns nurses to surgical cases. This procedure leads to confounding of team familiarity with the nurse manager’s assignment decisions, obscuring the true causal effect. To unearth this effect, addressing the selection bias inherent in the nurse manager’s assignment strategy is vital. This aspect represents a shortcoming in existing literature, where such confounding factors are not adequately addressed, resulting in studies that primarily reveal associations rather than causation.

Our paper aims to tackle this specific issue by using a structural causal modeling (SCM) framework. Employing this framework allows us to address confounding variables and estimate the implied causal relationships. Our findings suggest a statistically significant negative causal relationship, under our DAG assumptions, between the level of familiarity between the surgeon and the scrub nurse and the duration of the surgical procedure. When the surgeon and the scrub nurse are more familiar with each other, surgeries tend to be shorter. Hence, our study is, to the best of our knowledge, the first to estimate the effect of surgical team familiarity with a causal interpretation across an entire hospital’s surgical portfolio, extending beyond prior work focused on individual specialties or correlational evidence. In alignment with previous studies, we argue that a shorter duration of a surgical procedure serves as a proxy for increased efficiency in the surgical process [6, 46]. This underscores the importance of teamwork and familiarity in reducing the time it takes to complete surgical procedures, which can have positive implications for patient care and hospital efficiency.

The rest of this paper is organized as follows: Section 2 summarizes the relevant research in the literature. Section 3 introduces our data and metrics. Section 4 presents our causal model, model testing process, and results. Section 5 discusses the model results. Section 6 outlines the limitations of this study. Section 7 concludesAppendix A offers an overview of the SCM framework. Appendix B provides additional details about the model testing process. Appendix C includes different model specifications for sensitivity testing. Appendix D provides team familiarity and procedure duration statistics for the 25 most frequently performed procedures in our institution.

## Literature review

Prior research has recognized the significance of teamwork as a key factor driving performance across various industries, including domains like sports, aviation, and manufacturing [13, 25]. In the context of the operating room (OR), collaborative teamwork has been associated with notable improvements in several crucial aspects. These include enhanced surgical efficiency, decreased surgical errors and adverse events, improved overall quality of medical care, decreased waste and heightened safety levels during surgical procedures, as highlighted by studies such as [10, 14, 18, 24, 31, 32, 37, 51, 59, 60, 65] and [66]. What’s particularly intriguing is that there is evidence suggesting that effective teamwork within the OR may exert a more substantial influence in reducing surgical errors compared to the level of experience possessed by the surgeon [14]. Building on this understanding, recent studies continue to highlight critical factors influencing healthcare efficiency and outcomes. For instance, Zaranko et al. (2023) demonstrate the importance of registered nurse staffing levels and seniority for patient mortality. Similarly, Stecher and Ketcham (2023) show that shared experience among surgical teams significantly improves productivity and reduces costs without sacrificing clinical quality.

The fundamental elements of successful teamwork encompass individual and collective experiences, as well as a range of skills such as technical proficiency, decision-making abilities, adaptability, and interpersonal competence [9]. Effective communication is also a crucial aspect within these core domains [9]. When team members collaborate, it fosters the creation of shared mental models related to team cognition. This shared understanding, in turn, contributes to improved efficiency within the team [9]. In essence, a combination of individual and collective experiences, diverse skills, and effective communication forms the bedrock of effective teamwork, with collaborative experiences playing a key role in enhancing overall team efficiency through the development of shared mental models. Therefore, cultivating a sense of familiarity within a team is a pivotal aspect of enhancing teamwork [9, 21, 35].

Acknowledging the vital significance of teamwork, various efforts, including initiatives like TEAM-STEPPS, Six Sigma, and other process-oriented measures, have been implemented with varying degrees of success in ORs nationwide [3, 17, 38, 50, 63]. Another instance of optimizing team familiarity is observed in the consistent pairing of physicians and nurses in ambulatory settings, aimed at minimizing patient harm and enhancing communication within the healthcare environment [22, 26].

Previous studies on operating room teamwork have tended to be either case-specific or specialized to particular medical fields. These studies typically narrow their focus to specific interactions among team members, such as familiarity between surgeons [4, 19, 51], surgeon-nurse pairs [7, 10, 12, 16, 48, 65], or surgeon-trainee relationships [14, 32, 37, 66]. Researchers have less frequently broadened their team definition beyond these pairs to incorporate additional personnel [54, 55, 58]. Examining familiarity with the surgeon, some researchers isolate the experiences of nurses in the scrub and circulator roles [61].

Numerous studies have examined the relationship between team familiarity and performance, using various definitions of familiarity and focusing on specific surgical procedures or specialties [6, 14, 31, 37, 46, 54, 55, 65, 66]. For example, Avgerinos & Gokpinar (2017) analyze cardiac surgeries and demonstrate that higher team familiarity improves productivity, particularly under complex conditions. KC & Staats (2012) study how surgeons’ focal and related experience affects performance, highlighting the nuanced impact of experience accumulation across hospitals. While these and other studies provide important insights, they typically focus on correlational analyses within narrow clinical domains and do not attempt to identify causal effects.

Our study builds on this body of work by extending the scope to the hospital-wide surgical portfolio and employing a formal causal inference framework to estimate the implied effect of team familiarity on procedure duration. Unlike prior research that often relies on average familiarity measures within single surgical specialties, we control for potential confounders using staff fixed effects and apply a consistent methodology across all surgeries. Our decision to analyze team familiarity across the hospital’s entire surgical portfolio is driven by its crucial implications for both theoretical understanding and practical application. This comprehensive scope allows us to establish that team familiarity effects are not confined to specific procedures, but represent a generalizable phenomenon across diverse surgical settings. From a practical standpoint, understanding these effects at a hospital-wide level is essential for informing integrated management decisions, such as optimizing team assignment and scheduling across the entire surgical suite, thereby fostering system-wide improvements in efficiency and patient outcomes. This broader analysis also enhances the statistical power and reliability of our findings.

The methods used to measure familiarity among these individuals vary significantly. The most straightforward approach entails examining cases based on the frequency of previous team collaborations, using this as a surrogate measure for the level of familiarity between individuals [7, 14, 16, 37, 51, 66]. On the other hand, certain authors have opted to assess the structural arrangements of consistent team pairings, utilizing rotations or service structures to distinguish between teams with high and low levels of familiarity [12, 16, 32]. An alternative approach is to group team members according to how often they participate in cases within a particular medical specialty or collaborate with a specific surgeon in comparison to their total caseload. This method essentially measures the proportion of an individual’s time devoted to a specific specialty or collaborating with a particular surgeon [4, 65]. Indirect approaches to assess familiar and unfamiliar teams have included classifying familiar teams based on the case start time, distinguishing between those during regular day shift hours or within the surgeon’s typical block time [7, 58]. Even with significant administrative burdens, a small number of researchers have conducted case-control studies and explored the impact of maintaining consistent operating room teams throughout the day [54, 55].

Notably, despite differences in study design, prior studies consistently report time savings associated with increased team familiarity—typically on the order of minutes per pro-cedure—which aligns with the magnitude of effects we observe. Our findings, therefore, contribute to the growing consensus on the operational value of team familiarity, while offering a broader perspective strengthened by an assumed causal structure.

Although prior literature extensively studied the impact of familiarity among various surgical team members, there has been relatively limited attention given to understanding the unique contributions of nurses in different roles within the surgical team, specifically circulating nurses (who work in the operating room but are not in a sterile environment, often referred to as circulators) and surgical scrub nurses (who are part of the sterile field within the operating room, known as scrubs), the impact of their familiarity with the surgeon and with each other. In the limited literature available on this topic, [28] does suggest a positive impact of increased team familiarity among scrub nurses on surgical performance. Nonetheless, it’s worth noting that their study primarily focuses on assessing familiarity at the team level, without distinguishing the familiarity levels among individual team members.

In summary, the existing body of literature has shed light on the connection between teamwork and the duration of specific surgical procedures. However, it falls short in examining the broader impact of teamwork across an entire hospital’s surgical portfolio and lacks research that attempts to identify a causal relationship.

## Data and metrics

In this section, we discuss the dataset underpinning our study, which provides the history of surgical procedures conducted at a high-volume academic medical center spanning from January 2017 to Nov 2023. Subsequently, we introduce how we quantify the degree of familiarity within surgical teams, which plays a pivotal role in our goal to argue a causal relationship between team familiarity and surgery duration.

### Data sources, collection, and study population

Data covering all surgical procedures conducted from January 2017 to May 2023 in a large medical center were obtained from electronic medical records. The COVID-19 pandemic introduced increased staff turnover, which likely led to greater variability in team familiarity. This heterogeneity strengthens our analysis by providing a wider range of familiarity levels within the data. To the best of our knowledge, COVID-19 did not introduce any confounding factors that would alter the fundamental causal relationships we are modeling. These data were de-identified through an automated process facilitated by a data query. Working in collaboration with the research informatics center, this query was employed to establish a prospective data repository.

### Procedure characteristics

The surgical records include case date and time, case timestamps, clinical service line (SL) / specialty, primary attending surgeon and nursing staff involved, and procedure type, which is defined by the unique numerical codes used primarily to identify primary procedure listed in the electronic health record (e.g. foot amputation, anterior total hip arthroplasty) and roughly approximates the Current Procedural Terminology (CPT) codes. The medical records also identify a main attending surgeon in situations involving multiple attending surgeons. Moreover, we extracted whether the procedures involved any use of robots to identify robotic and nonrobotic cases. Cases with incomplete data were excluded.

To understand the impact of teaming, we analyzed a total of 33,374 surgical procedures from a wide range of specialties including cardiac surgery, orthopedic surgery, neurosurgery, thoracic surgery, urology, gynecology, transplant surgery, general surgery and colon & rectal surgery. There are 890 different types of procedures performed in our institution.

We included only nonrobotic cases as robotic procedures require additional nurse training and certification and the use of robots can impact the procedure duration in a non-systematic way. We excluded cases that include multiple procedures (multipanel cases) as our data is not conducive to calculating the duration of each procedure involved in the case separately. We included both elective and urgent/emergent cases.

The nurse manager responsible for the operating room ensures the availability of at least one room and surgical team continuously for emergent trauma cases. Another room is kept ready for urgent add-ons, and both are staffed independently of the planned elective case schedule. Initial assignments for elective cases are made the day before, but adjustments can be made on the same day in case of cancellations or staff call-offs.

### Team characteristics

According to institutional policy, scrub technicians are restricted from serving as circulators, while registered nurses have the flexibility to perform either role within a single case. For simplicity, both technicians and nurses will be collectively referred to as “nurses” henceforth. Additionally, we will use “service line” and “specialty” interchangeably. Team analyses excluded residents, fellows, and physician assistants.

In each case, the primary circulator and scrub nurse were identified as the individuals who spent the most time in their respective roles. In case of a tie, the nurse documented as present at the earliest time was designated as the primary.

To ensure a sufficient number of observations in each cell, we excluded cases involving pairs with less than 5 interactions. The resulting data set involved 180 attending surgeons, 219 scrub nurses and 188 circulator nurses.

### Procedure duration

In our study, we characterize the *procedure duration* as the time span from when a patient enters the operating room (OR) to when they leave, i.e. “wheels in to wheels out”. We selected this specific metric because it directly aligns with hospital scheduling and staffing practices; per JCAHO guidelines, this entire duration mandates the continuous presence of the operating room technician/nurse, circulating nurse, attending anesthesiologist, and surgeon. We found "wheels in to wheels out" to be a more accurate and reliably documented measure compared to "case start time." The latter is often inaccurately recorded due to the circulating nurse’s numerous tasks at the beginning of a case, which can delay their ability to precisely mark the start. Furthermore, the initial surgical team is typically present for the entire case, especially during the reproducible timeout, which serves as a consistent marker for the "wheels in" period. At the University of Chicago, where our data was gathered, while anesthesiology technicians may assist with intubation and residents with patient positioning, we believe that staff familiarity continues to influence the efficiency of these preparatory tasks, particularly those relying on the circulating and scrub nurses.

**Fig. 1:**
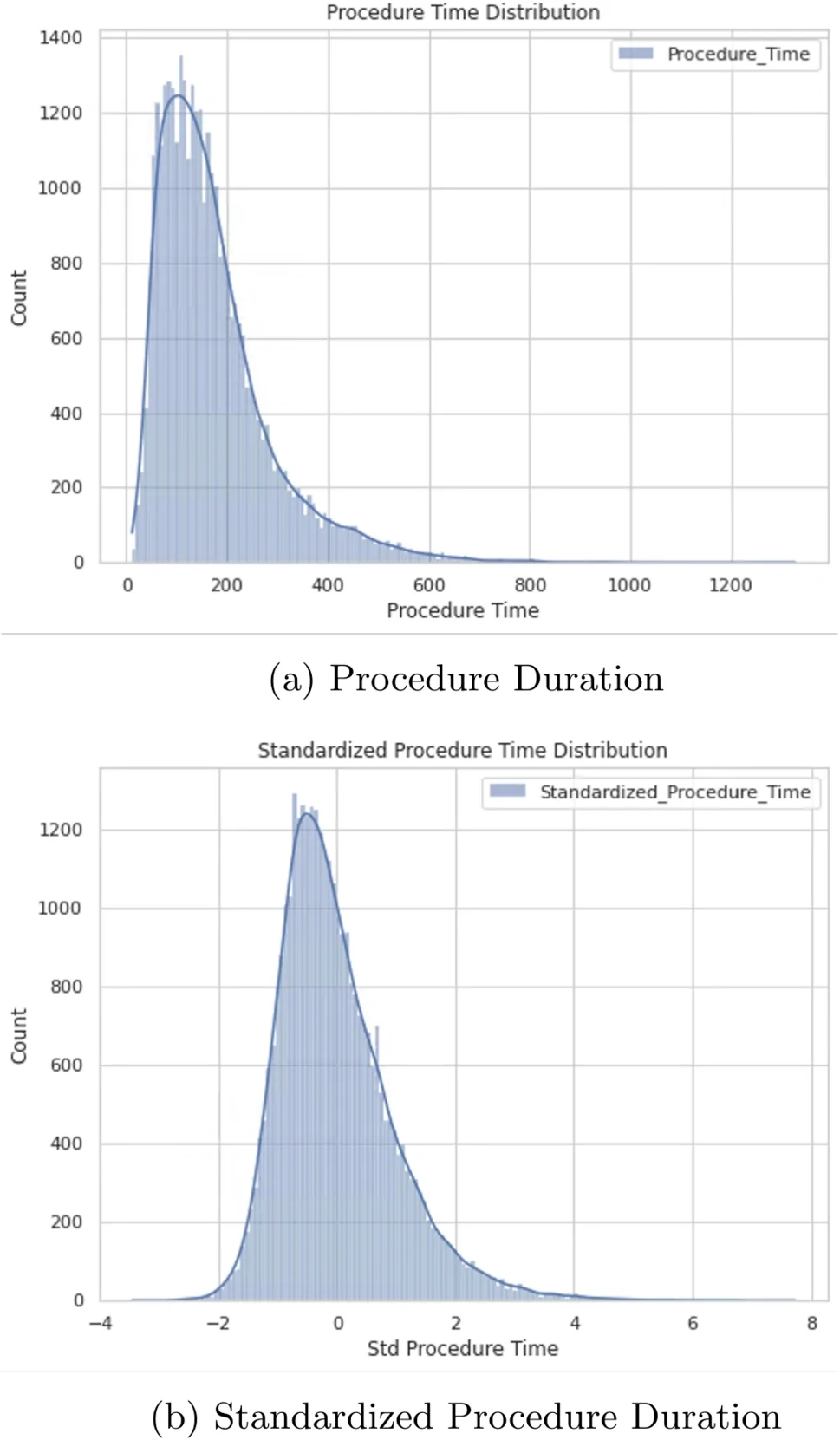
Procedure time distribution before and after standardization

Our primary motivation for investigating procedure duration extends beyond its direct impact on efficiency. We contend that decreased procedure duration in the OR is associated with notable improvements in several crucial aspects, including decreased surgical errors and adverse events, improved overall quality of medical care, decreased waste, and heightened safety levels during surgical procedures. Moreover, we view procedure duration as a critical proxy for team satisfaction, a known driver of significant organizational benefits. Prior research, such as Witmer et al. (2024), has established a strong link between procedure duration and team satisfaction, noting that longer-than-expected procedure times were significantly associated with worse team satisfaction [62]. In the current healthcare landscape, with the prevalent issue of physician and healthcare worker burnout, enhancing team satisfaction has become an organizational imperative. By identifying factors that contribute to efficient and predictable procedure durations, we are, by extension, addressing key determinants of team well-being and reducing burnout potential.

**Fig. 2:**
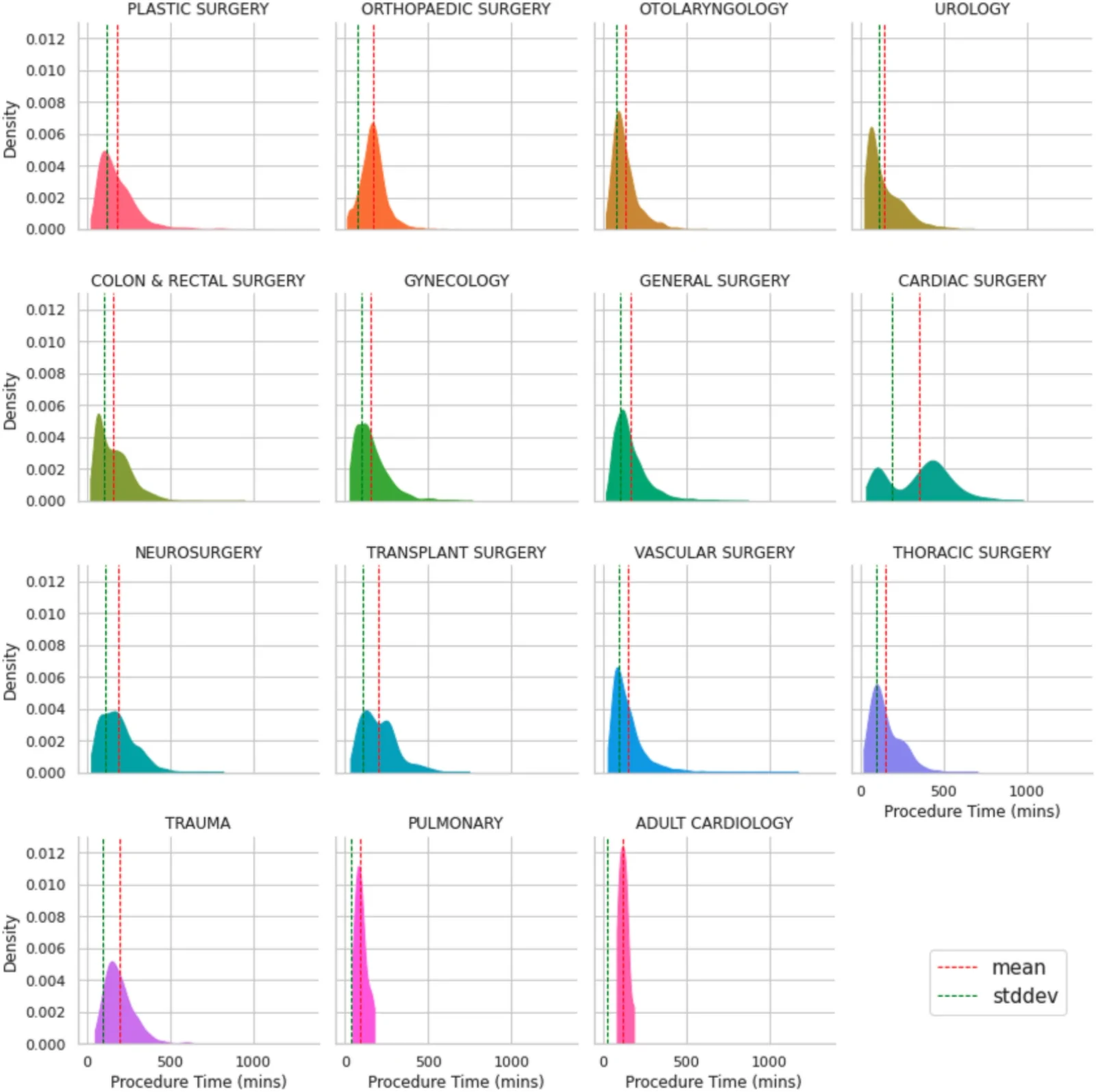
Procedure time distribution by specialty

Beyond its link to team satisfaction and broader OR improvements, focusing on procedure duration is also justified by its robust measurement. Its nature as a well-defined and accurately measured statistic for every single case makes it a highly reliable signal for analysis. This inherent accuracy and universal applicability make it an excellent proxy for understanding underlying team dynamics and their impact. While it is challenging to isolate the specific carryover benefits to patient outcomes directly from small changes in duration, the established link between teamwork (reflected in efficient procedures) and these broader positive outcomes reinforces the importance of our investigation. Additionally, benefits such as potential reductions in material waste further underscore the broader managerial relevance of understanding and optimizing procedure duration

**Fig. 3:**
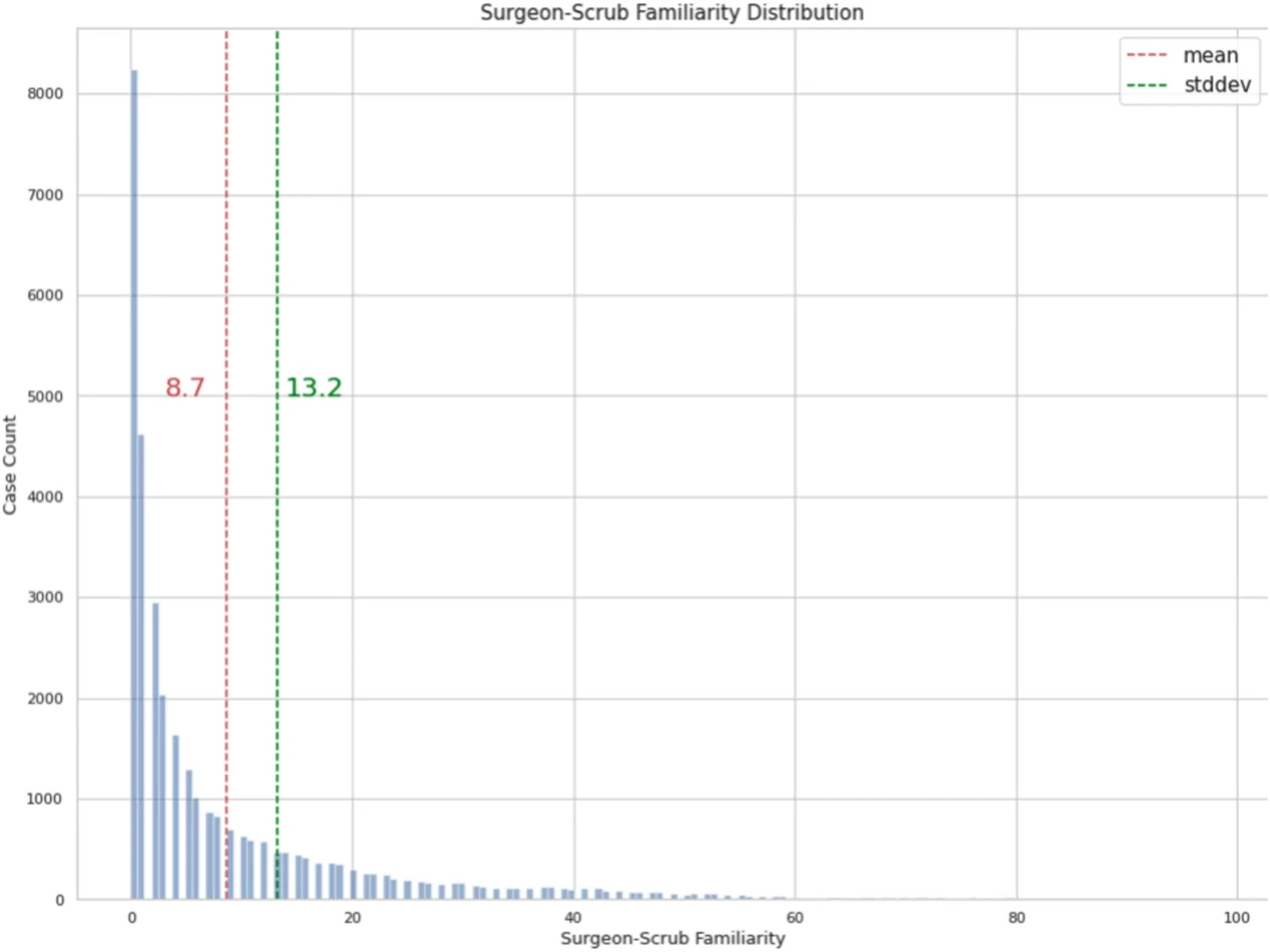
Surgeon-scrub familiarity distribution

**Fig. 4:**
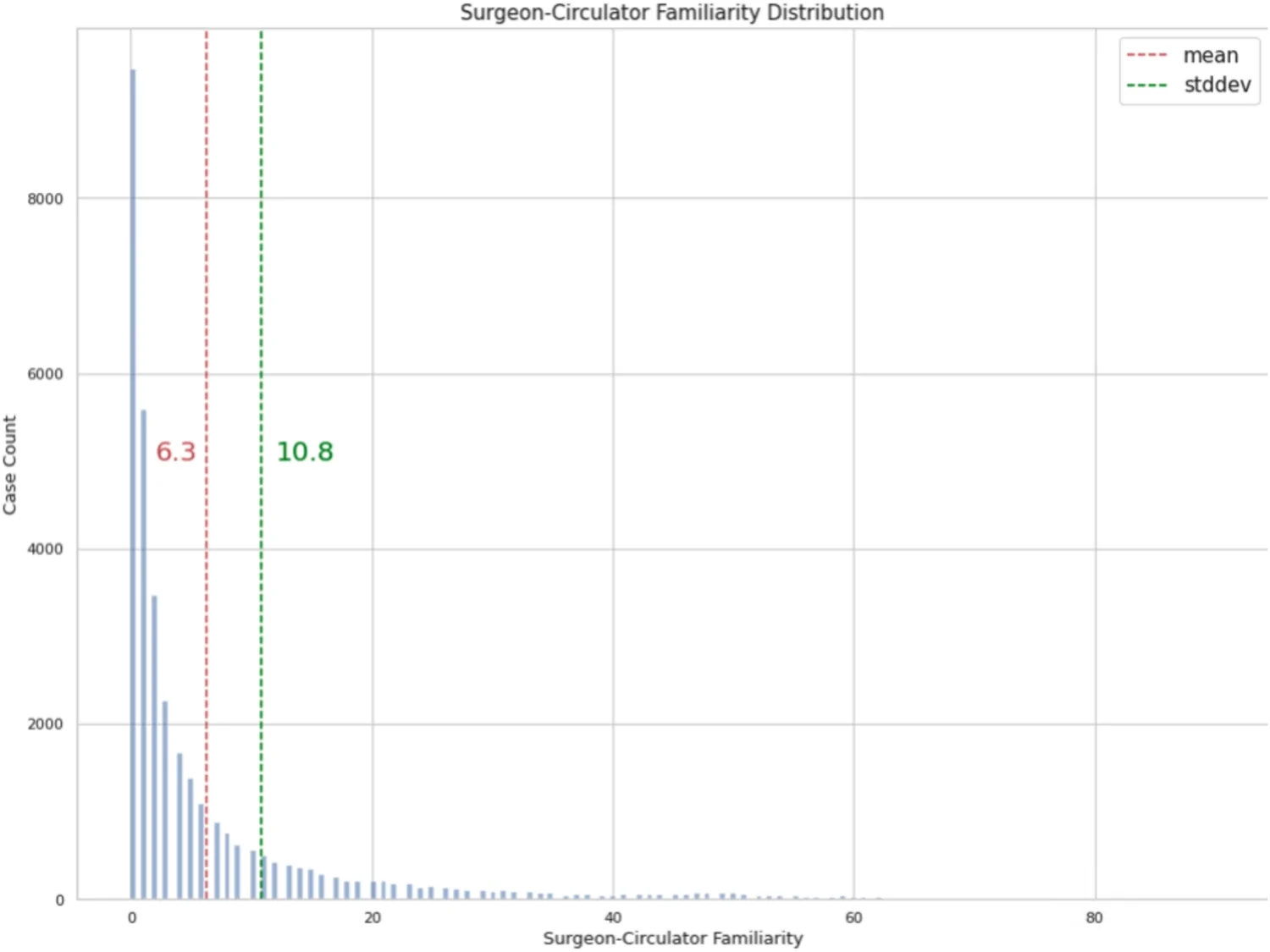
Surgeon-circulator familiarity distribution

We observe that the duration of procedures varies significantly across cases with different complexities, as illustrated in Fig. 1. Additionally, even within the same service line, procedures can exhibit substantial differences in duration, see Figure 2 where red dashed lines represent the mean procedure duration in each service line. Including a wide array of surgical procedures with different nature and complexity in our study gives rise to the need to standardize the covariates to account for variations across different types of procedures and specialties. Thus, we define the *standardized procedure duration*:

$$\begin{aligned} t_{std} = \frac{t- \mathbb {E}[t]}{\sigma [t]}, \end{aligned}$$

where $\mathbb {E}[t]$ and σ[t] are taken with respect to the procedure type.

This standardization also allows us to make counterfactual statements such as teaming reduces standardized times by .1 standard deviations, which can be generalized across surgical cases and surgical centers.

**Fig. 5:**
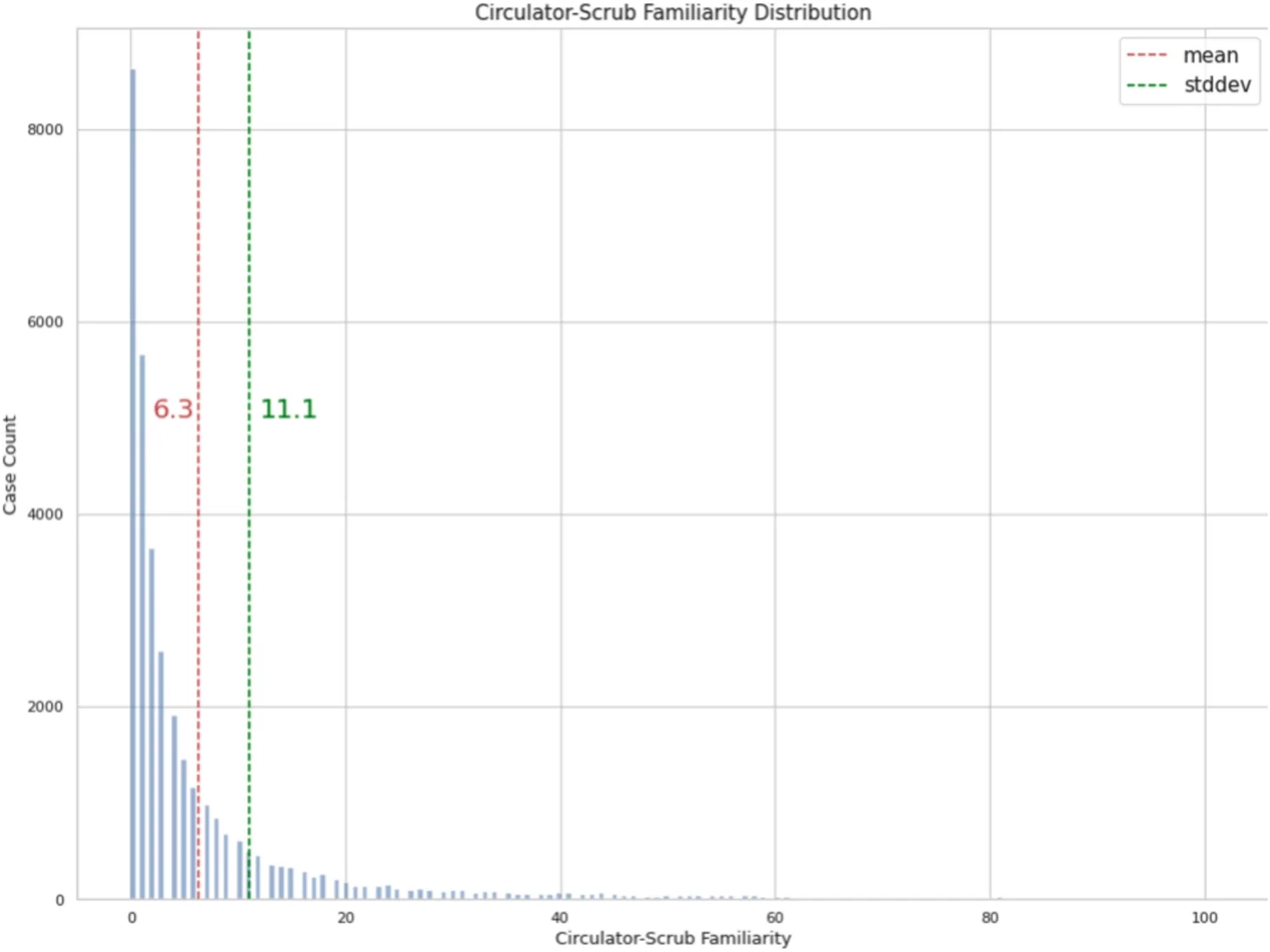
Circulator-scrub familiarity distribution

**Fig. 6:**
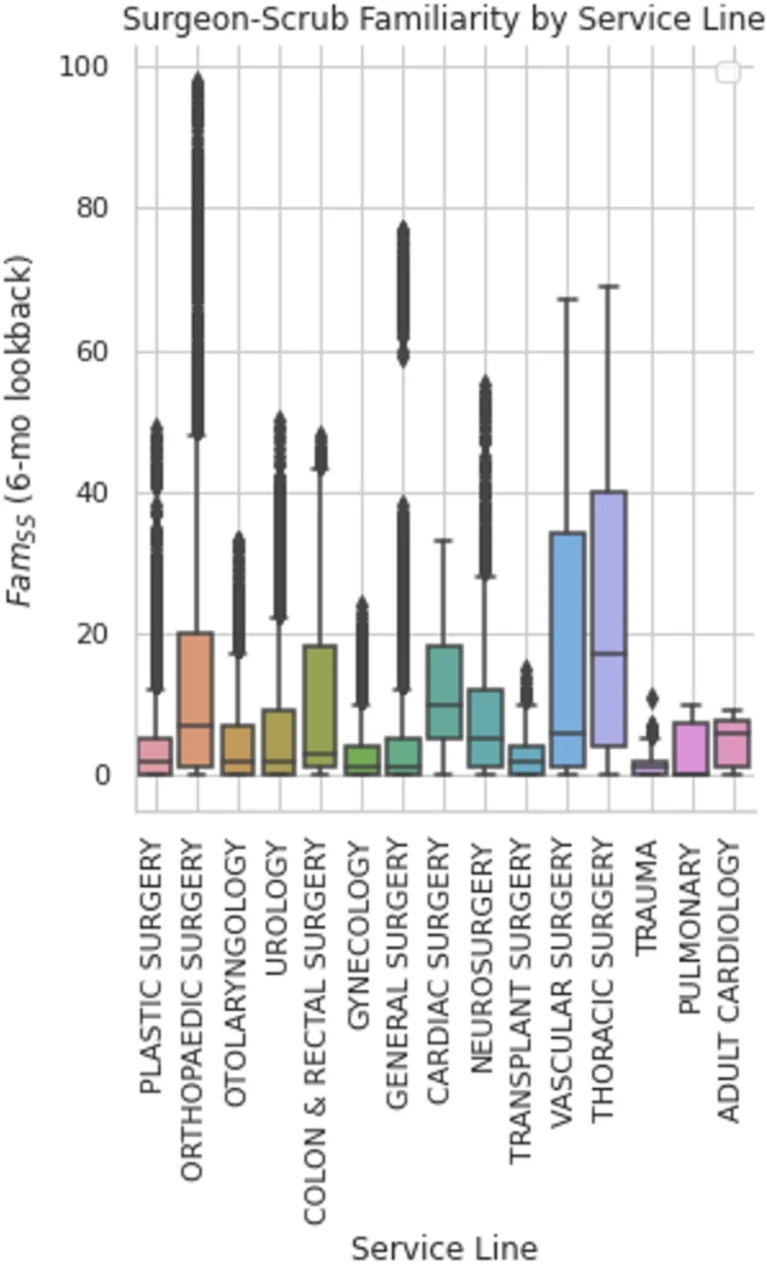
Surgeon-scrub familiarity

### Team familiarity metrics

We define the *team familiarity* (*Fam*) for a given pair of surgical team members (surgeon-scrub nurse, surgeon-circulator nurse, circulator-scrub nurse) as the number of cases on which the pair worked together in the past 6 months. The distribution for each statistic are presented in Figs 3, 4 and 5, respectively. We make the same calculation for 9 months and 12 months in addition to 6 months to make sure our results are robust to the different lookback periods. While calculating the team metrics for an n-month lookback period, we exclude cases that occurred within the first n months of the data collection start date. This exclusion was done to minimize bias in teaming caused by team members with very low procedure volumes and newly hired staff.

**Fig. 7:**
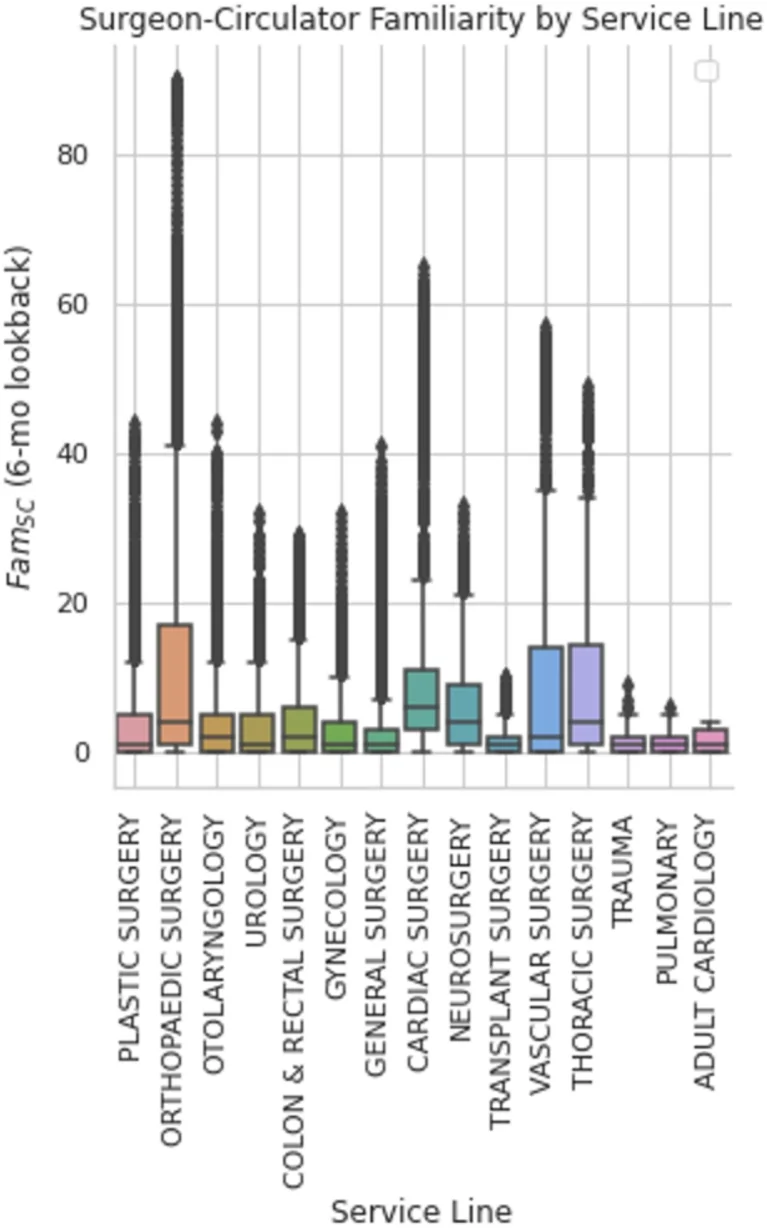
Surgeon-circulator familiarity

**Fig. 8:**
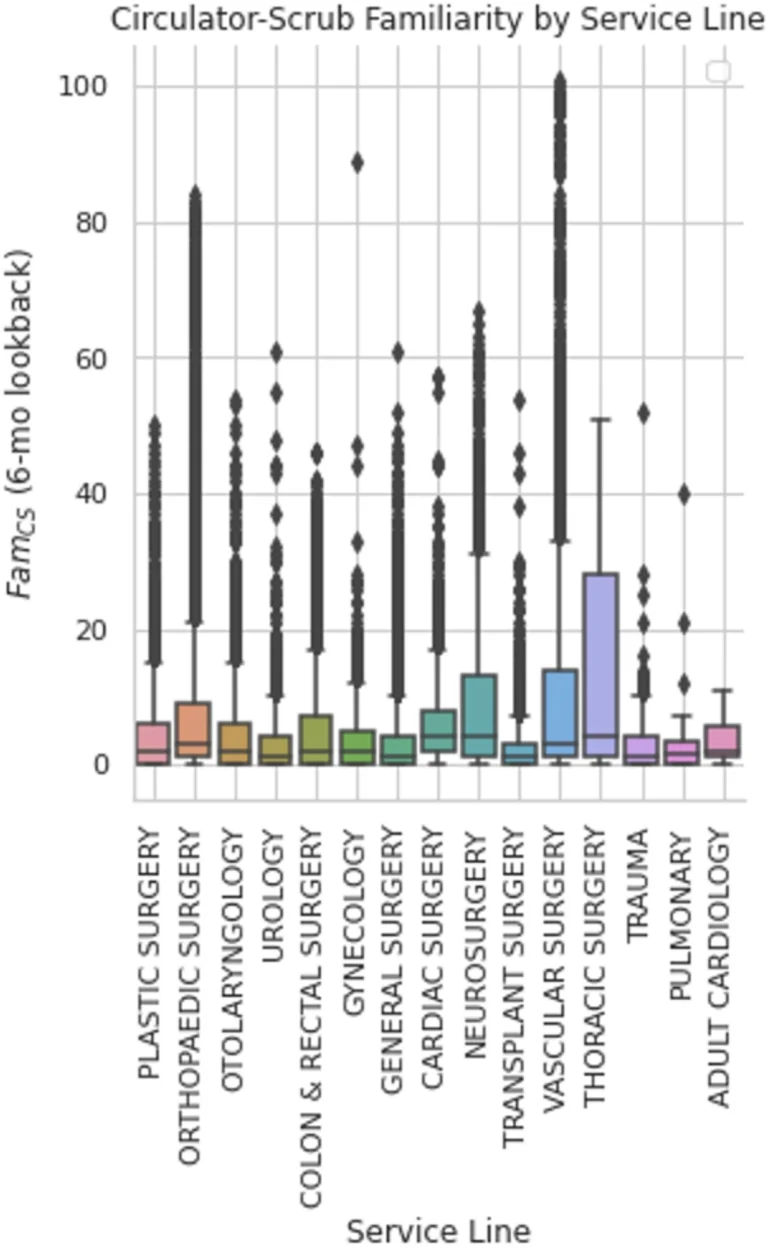
Circulator-scrub familiarity

We define the *standardized surgeon-scrub familiarity* as

$$\begin{aligned} Fam^{SgSc}_{std} = \frac{Fam^{SgSc} - \mathbb {E}[Fam^{SgSc}]}{\sigma [Fam^{SgSc}]}, \end{aligned}$$

where $\mathbb {E}[Fam^{SgSc}]$ and $\sigma [Fam^{SgSc}]$ are taken with respect to the procedure type. This standardization allows us to control for different levels of team familiarity across service lines and different procedure types as our data analysis reveals varying degrees of teamwork across distinct service lines, see Fig. 6. If some team members frequently attend procedure types that take longer, this leads them to accumulate less team familiarity in terms of case counts and create bias in the team familiarity metric. Standardization by procedure type addresses this issue as well. Moreover, given that we standardize the procedure duration with respect to the procedure type, we use the same standardization approach to have comparable units across all covariates.

Similarly, we define the *standardized surgeon-circulator familiarity* as

$$\begin{aligned} Fam^{SgC}_{std} = \frac{Fam^{SgC} - \mathbb {E}[Fam^{SgC}]}{\sigma [Fam^{SgC}]}, \end{aligned}$$

where $\mathbb {E}[Fam^{SgC}]$ and $\sigma [Fam^{SgC}]$ are taken with respect to the procedure type (Fig. 7). We define the *standardized circulator-scrub familiarity* as

$$\begin{aligned} Fam^{CSc}_{std} = \frac{Fam^{CSc} - \mathbb {E}[Fam^{CSc}]}{\sigma [Fam^{CSc}]}, \end{aligned}$$

where $\mathbb {E}[Fam^{CSc}]$ and $\sigma [Fam^{CSc}]$ are taken with respect to the procedure type (Fig. 8). Team familiarity metrics are calculated on a case-by-case basis and they are not global metrics. In other words, for a given surgical case, we calculate the team familiarity of the surgical team members who work on that particular case. These metrics serve as covariates in our causal model.

### Service line experience metrics

**Fig. 9:**
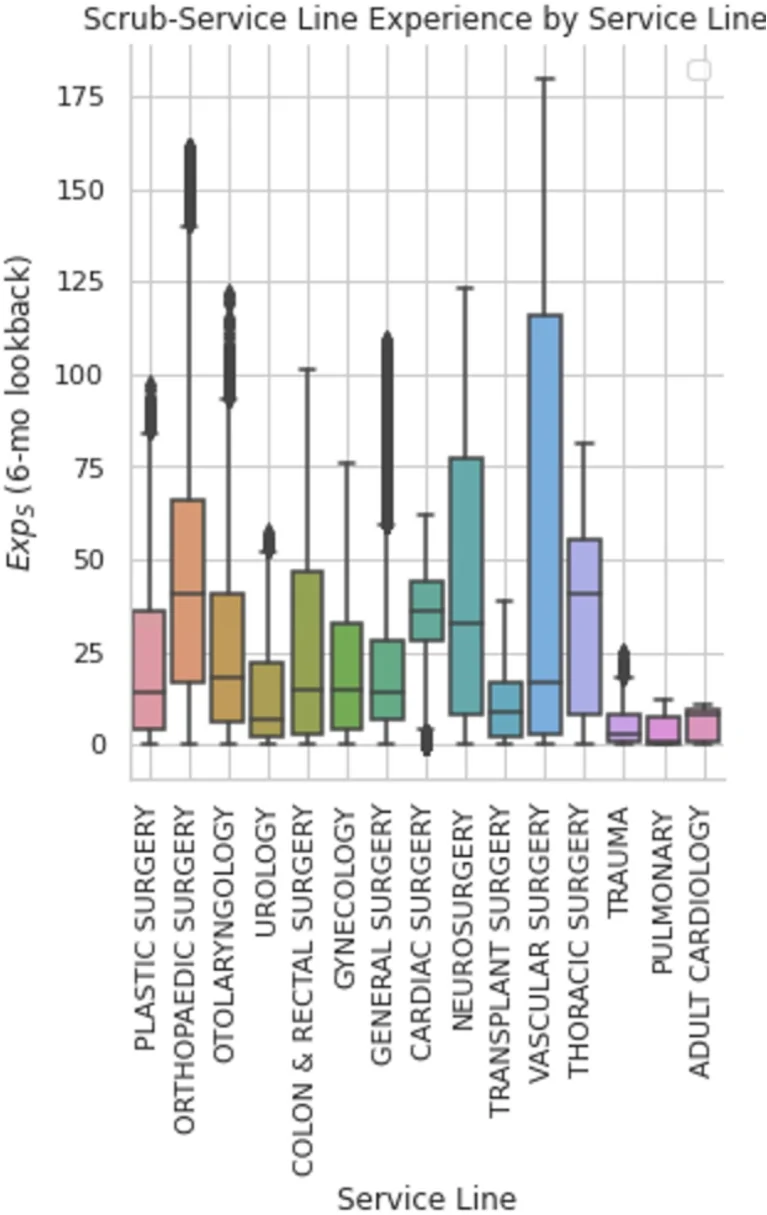
Service line experience of scrub nurses by service line

Nurses frequently find themselves assigned to cases in multiple service lines, engaging in collaborative efforts with a multitude of surgeons. This situation presents a considerable opportunity to form surgical teams in diverse configurations. Thus, it is important in our analysis to take into account the experience levels of the surgical team members within a specific service line because their expertise is likely to affect how long a procedure takes. To that end, we define the *service line experience* (*Exp*) as the staff caseload in the specialty of the case excluding the collaborations with the assigned team members in the past 6 months^2^. This exclusion is crucial as it allows *Exp* to capture an individual’s generalizable service line experience, distinct from recent collaborative experiences with the current team, which are accounted for by separate covariates in our model. This approach prevents double-counting collinear components of experience and enables a clearer interpretation of the unique contributions of individual service line experience and team familiarity. To elaborate, consider a scenario where Nurse A (scrub), Nurse B (circulator) and Surgeon A are assigned to a given cardiac case. Nurse A and Surgeon A collaborated 30 times in the past 6 months on other cardiac cases. Nurse A and Nurse B never collaborated in the past 6 months. Hence, $Fam^{SS}$ associated with this cardiac case is equal to 30, and $Fam^{CS}$ is 0. Furthermore, assume Nurse A worked on a total of 75 cardiac cases in the past 6 months. Given that 30 of those were with Surgeon A and counted in the familiarity metric, the service line experience metric for Nurse A for this surgical case is calculated as 45 (75 - 30). Thus, *Exp* captures the experience of the surgical team members in the service line of the case with other surgeons and nurses (Fig. 9).

**Fig. 10:**
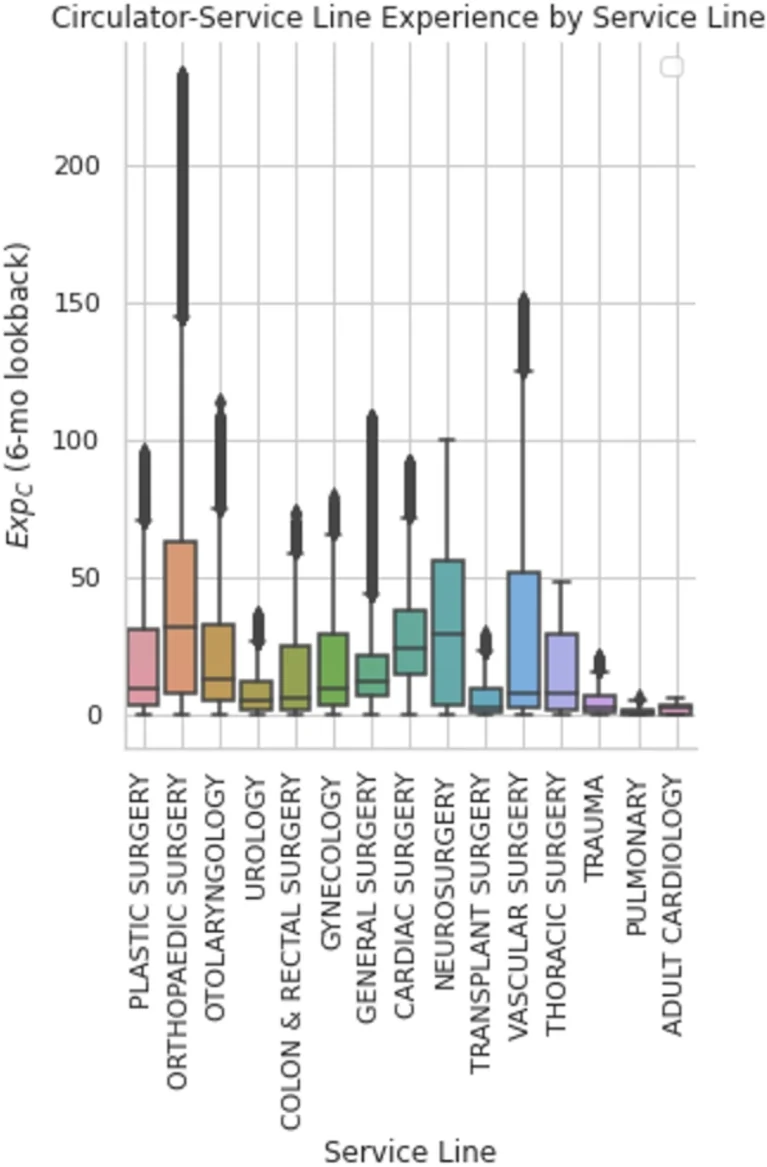
Service line experience of circulator nurses by service line

We define the *standardized service line experience* as

$$\begin{aligned} Exp_{std} = \frac{Exp - \mathbb {E}[Exp]}{\sigma [Exp]}, \end{aligned}$$

where $\mathbb {E}[Exp]$ and σ[Exp] are taken with respect to the procedure type. Standardizing in this way helps us account for the varying levels of experience among nurses in different service lines and with various types of procedures. Our data analysis indicates significant differences in service line experience across unique service lines, as illustrated in Fig. 10. Furthermore, since we’ve standardized both the duration of the procedures and the familiarity metrics relative to the type of procedure, it’s crucial to apply a consistent standardization method. This ensures that all covariates are measured in comparable units. We calculate this metric for the surgeons, scrub nurses and circulator nurses separately. We denote these as $Exp_{std}^{Sg}$, $Exp_{std}^{Sc}$ and $Exp_{std}^{C}$, respectively.

## Causal model and results

Within this section, we embark on a comprehensive exploration of a causal relationship between team familiarity and surgery duration, under the assumptions given by a DAG model. When applying statistical methods to argue a causal relationship based on observational data, one attempts to avoid biased estimates among the variables under study, due to issues like confounding variables, overcontrol, and collider bias.

We acknowledge that alternative empirical strategies, such as those employing quasi-experimental methods, have been successfully utilized in related literature to establish causal estimates when randomization is not feasible. For instance, Chen (2021) leverages specific institutional features, including the quasi-random assignment of patients to doctor teams with varying shared work experience through unanticipated emergency department admissions. Similarly, Kelly et al. (2023) exploit the daily rotation of nursing staff across teams and within-ward variation in staffing levels, complemented by rigorous tests for endogeneity, to identify causal effects. These quasi-experimental designs, while powerful, rely on the presence of specific exogenous variation in the exposure of interest (e.g., team familiarity or composition) that is plausibly unrelated to unobserved patient or team characteristics. Our dataset, unfortunately, does not possess these specific sources of plausibly exogenous variation or natural experimental conditions that would allow for the direct application of such quasi-experimental methods.

**Fig. 11:**
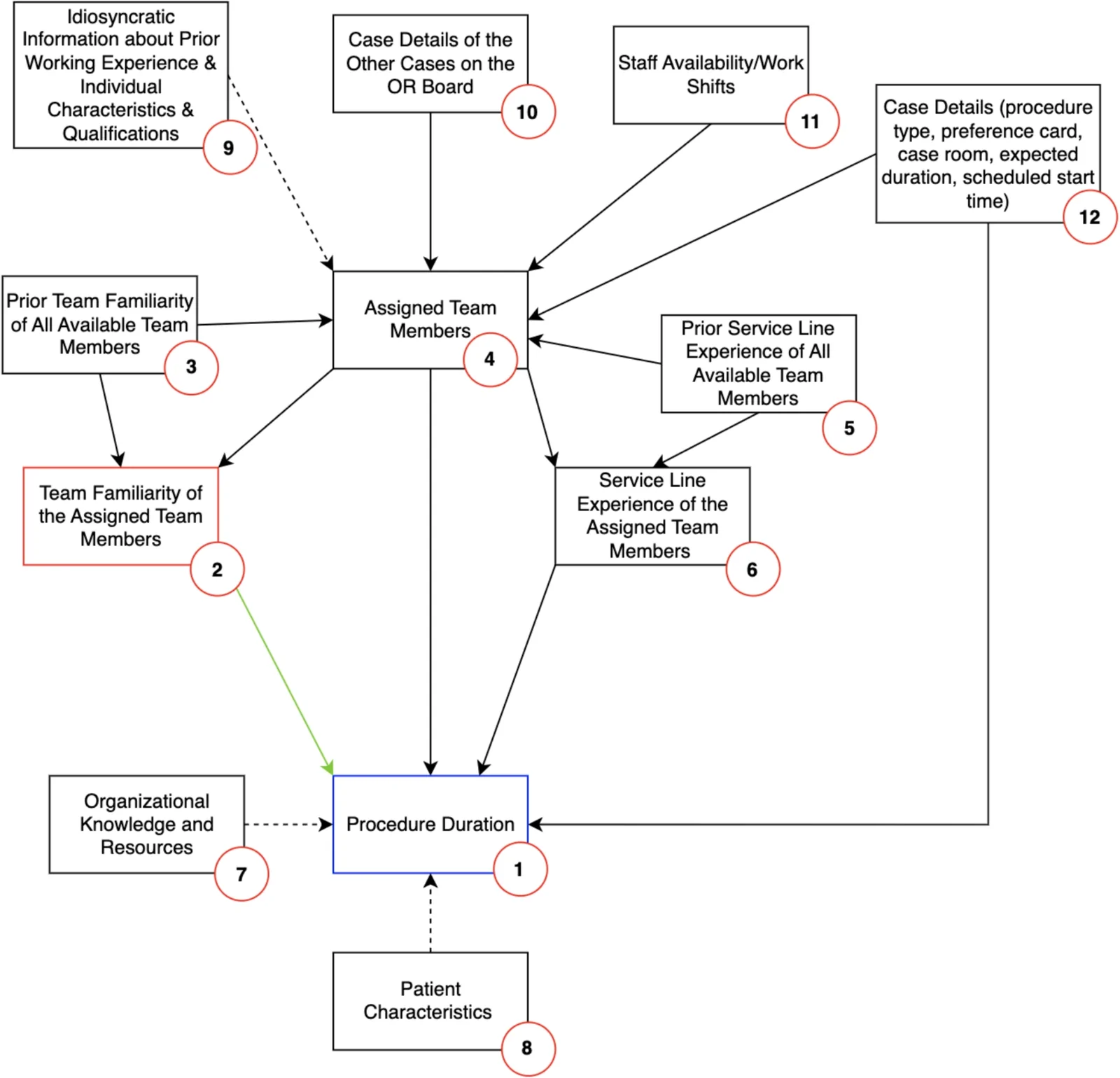
Causal Forces Affecting the Procedure Duration. Outcome and treatment variables are highlighted in blue and red, respectively; the causal relationship of interest is marked by the green arc; the causal impact of the variables that are unobservable in our data are marked by dashed lines. The variables are marked by numbers for ease of reference and numbering does not communicate any information

Therefore, to address the aforementioned challenges and to rigorously investigate causal relationships within the constraints of our empirical setting, we use structural causal modeling (SCM). SCM is designed to help identify cause-and-effect relationships from observational data, offering a solution to the limitations of traditional statistical adjustments.

The Structural Causal Modeling (SCM) framework em-ploys Directed Acyclic Graphs (DAGs) to visualize and analyze the relationships between variables, particularly focusing on causal relationships. Subsequently, a graphical rule based on DAGs, known as the *backdoor criterion*, is utilized to ascertain the necessary statistical adjustments (or the absence thereof) for arguing causal relationships from observational data. By utilizing this criterion, we can determine the appropriate set of variables Z within the causal model depicted by a DAG on which to condition when searching for a causal relationship between any two variables, X and Y. We present an overview of the SCM framework in Appendix A.

The estimated effect can be interpreted as causal if the assumptions represented in the DAG are correct. As is typical in observational studies, these assumptions cannot be fully verified using the observed data.

In this section, we outline the construction of our structural causal model using a DAG, establishing the theoretical framework that underpins our understanding of the causal pathways between team familiarity and surgery duration. Following the model’s formulation, we validate our proposed DAG using data. This involves testing the implications of the DAG in the form of causal independence statements. Subsequently, we utilize the *backdoor criterion* to identify the set of variables to adjust for in our statistical model to estimate the causal effects of interest. Finally, we report the estimation results obtained using ordinary least squares (OLS) regression.

### Causal model

Informed by the contextual insights provided by our collaborators within the Department of Surgery, we have synthesized the causal influences impacting the duration of medical procedures^3^ into a structured causal graph, also known as a directed acyclic graph (DAG), visually represented in Fig. 11. The variables in the figure are marked by numbers for ease of reference and numbering does not communicate any information. Our primary focus is directed towards attempting to isolate the causal impact of team familiarity on procedure duration, symbolized by the green arrow in the graph.

At our medical institution, the nurse manager manually assigns nurses to surgical cases in a non-random manner. Consequently, the effect of team familiarity is confounded with the nurse manager’s assignment process and isolating the causal effect requires addressing the selection bias introduced by the nurse manager’s assignment process. To tackle this bias, we analyze the factors influencing her decision-making process. The nurse manager considers the availability of nurses on a given day (node 11) while making the assignment decisions.

The nurse manager considers case details (node 12) such as the surgeon of the case, procedure type, preference card^4^, the room that the case is assigned to, expected duration of the case and scheduled start time while making the nurse assignments. These case details also affect the procedure duration directly in addition to affecting the assigned team members. As there can be many procedures taking place in parallel on a given day, the nurse manager takes the case details and the staffing needs of those other cases on the OR board (node 10) into account while deciding which nurses to assign for a given case.

The nurse manager might have idiosyncratic information about surgeons and nurses (node 9), which affects the assignments but is unobservable in our data. Finally, patient characteristics as well as organizational knowledge and resources of the institution affect the procedure duration, but these are unobservable to us. Importantly, the nurse manager generally does not use patient characteristics in making assignments.

Familiarity of the assigned nurses with the surgeon (node 2) and the service line experience of the assigned nurses (node 6) can play a role in how long the procedure takes. Assigned nurses (node 4) and case details (node 12) can also directly impact the procedure duration through factors other than their team familiarity (node 2) and service line experience (node 6). In Fig. 11, prior team familiarity (node 3) and prior service line experience of all available nurses (node 5) are global variables and are calculated based on historical data. Whereas, familiarity and service line experience of the assigned nurses (nodes 2 and 6, respectively) are case-specific and refer to the familiarity and experience of team members assigned to the specific surgical case at hand.

**Fig. 12:**
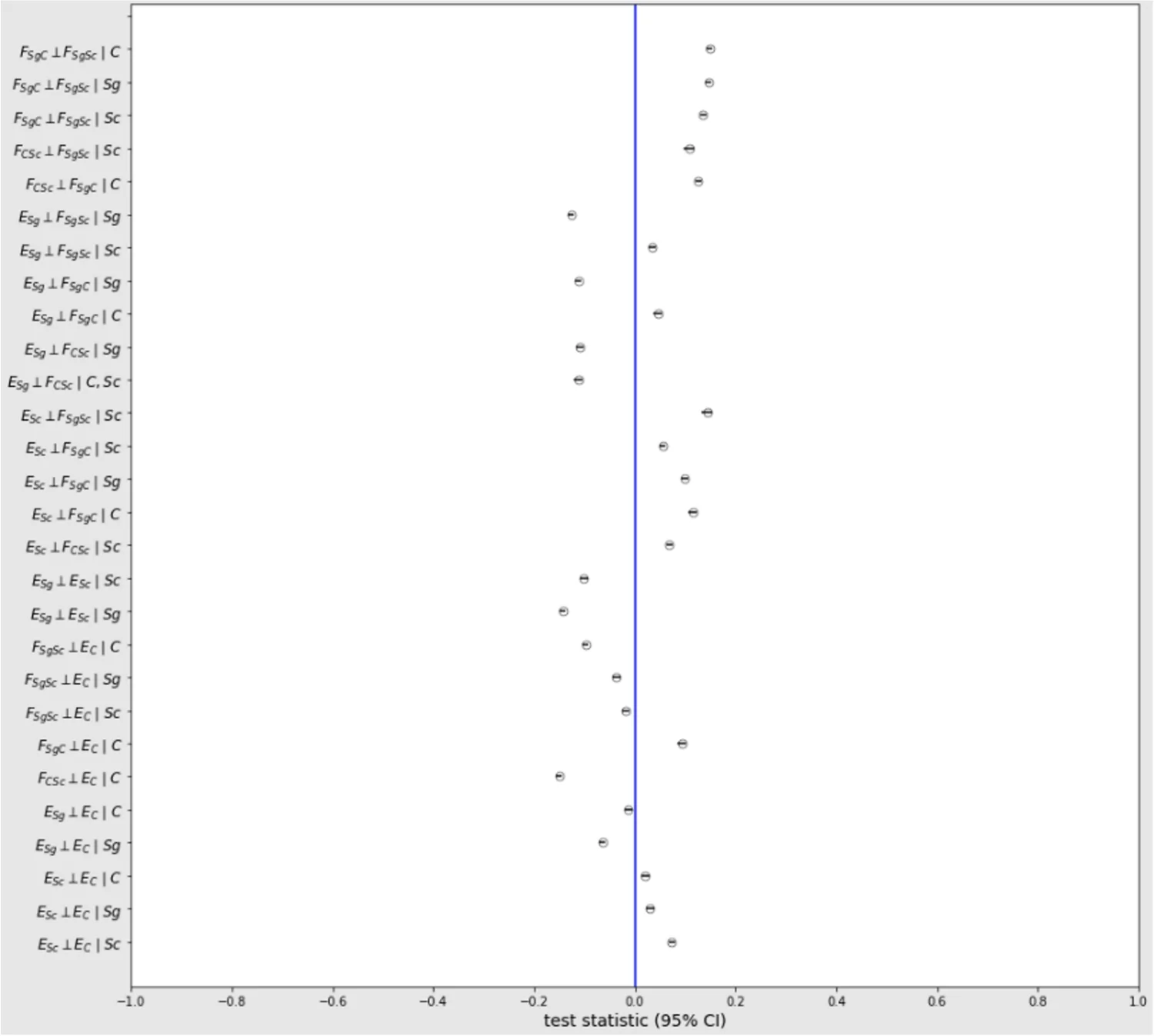
Conditional independence test results

To summarize, “case details” (node 12), “assigned nurses for the case” (node 4), “team familiarity of the assigned nurses” (node 2), “service line experience of the assigned nurses”(node 6), and “patient characteristics” (node 8) are case-specific variables that affect the “procedure duration” (node 1) of a given surgical case. The remaining variables are global.

By the principles of the SCM framework, we search for a set of observed variables that fit the backdoor criterion to identify the causal impact of team familiarity on procedure duration. An examination of the DAG in Fig. 11 shows that the backdoor paths that create noncausal pathways between familiarity and procedure duration are as follows:

$$\begin{aligned}&2 \longleftarrow 3 \longrightarrow 4 \longrightarrow 1, \\&2 \longleftarrow 3 \longrightarrow 4 \longrightarrow 6 \longrightarrow 1, \\&2 \longleftarrow 3 \longrightarrow 4 \longleftarrow 5 \longrightarrow 6 \longrightarrow 1, \\&2 \longleftarrow 3 \longrightarrow 4 \longleftarrow 12 \longrightarrow 1, \\&2 \longleftarrow 4 \longrightarrow 1, \\&2 \longleftarrow 4 \longrightarrow 6 \longrightarrow 1, \\&2 \longleftarrow 4 \longleftarrow 5 \longrightarrow 6 \longrightarrow 1, \\&2 \longleftarrow 4 \longleftarrow 12 \longrightarrow 1. \\ \end{aligned}$$

The variable “assigned nurses for the case” (node 4), which is not a descendant of “familiarity of the assigned nurses” (node 2) — our treatment variable — blocks all the backdoor paths. Therefore, “assigned nurses for the case” (node 4) satisfies the backdoor criterion. It’s important to note that a key requirement for applying the backdoor criterion is the observability of variables that close backdoor paths, allowing for their incorporation into our statistical model. Our DAG includes some unobservable elements, indicated by dashed arrows, but these do not create backdoor paths that necessitate control. Therefore, the backdoor criterion is applicable in our case. Should this not be the case, the “frontdoor criterion” would need to be employed. Since our DAG does not require this, we will not delve into this topic, but those interested may refer to Pearl’s works from 2009 [44] and 2016 [45].

As long as the true causal story is consistent with the DAG in Fig. 11, controlling for “assigned team members” for the case in our statistical model can be likened to the concept of “closing the backdoor paths” [45] and gives us the causal effect of team familiarity on procedure duration. This can be estimated from our observational data as the team members are recorded in our data. Adjusting for the assigned team members effectively neutralizes any non-causal factors that could potentially mediate the relationship between team familiarity and procedure duration. Before we move on to the estimation task, we validate our model and check if our DAG structure conforms to the underlying dataset in the following subsection.

### Model testing

The testable implications of DAGs are expressed through statements of conditional independence [57]. When statistically testing these independencies, the goal is to demonstrate that the variables under consideration are uncorrelated, potentially after conditioning on certain other variables. However, the specific tests employed vary based on the characteristics of the available data [1].

**Fig. 13:**
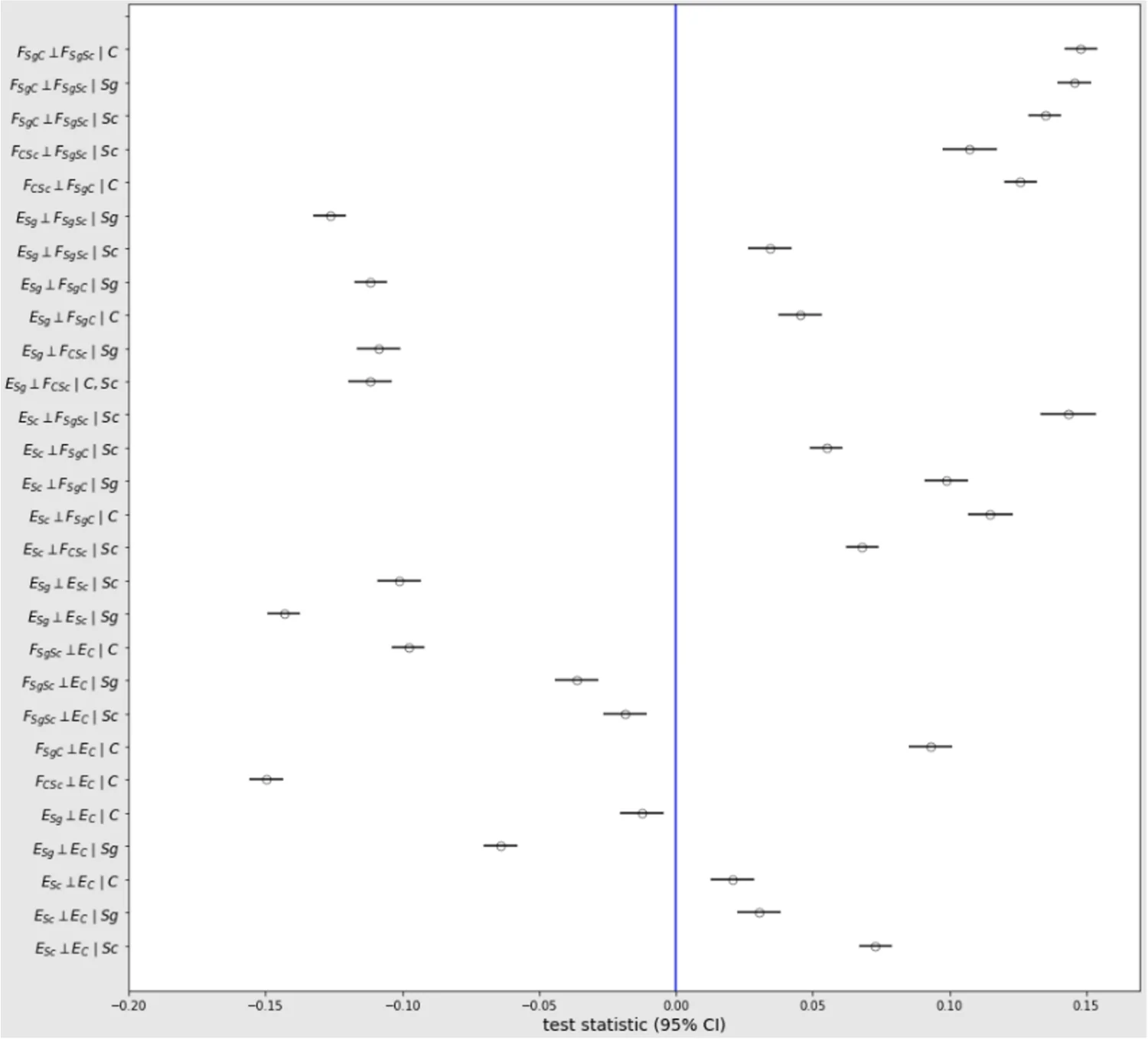
Conditional independence test results (Zoomed in)

The details of the conditional independence statements that are implied by our DAG model structure (Fig. 11) can be found in Appendix B. As the evaluation of the conditional independence statements posited by our DAG requires testing the independence of two continuous variables conditional on categorical variables (specifically, staff fixed effects), we can use partial correlation to test these statements. Given that partial correlation encapsulates the magnitude of the effect within a multiple regression framework while controlling for certain variables, we execute multiple linear regressions for each conditional independence statement. The results, inclusive of the point estimate of the effect sizes and their respective 95% confidence intervals, are presented in Figs. 12 and 13.

To illustrate, for a conditional statement such as $Y \perp X \,|\, Z$, we conduct a regression of *Y* on *X* and *Z* and subsequently report the 95% confidence interval pertaining to the coefficient of *X*. We expect test values to be around zero if the conditional independence is satisfied and underlying data aligns with the DAG structure. However, the test statistic is rarely equal to zero in reality due to random noise in the data. This can be the case even if the data is simulated based on the DAG structure [2].

In Fig. 12, our test statistic values are observed to be close to zero. Although they are not exactly 0, correlations of this size are considered negligible [36, 39, 42]. Hence, the violations of independence implications of this size are considered modest and their dependence is considered nearly zero [1, 5, 47]. Thus, our DAG structure offers a credible depiction of the underlying dataset, and causal arguments derived from this model can be considered plausible.

The next subsection includes results from various model specifications and robustness checks for three different lookback periods, namely 6, 9, and 12 months.

### Estimation and results

We employ ordinary least squares (OLS) regression to estimate the impact of teaming between surgeons and scrub nurses (SgSc), surgeons and circulator nurses (SgC), and circulator and scrub nurses (CSc). The general structure of our causal model specifications is outlined as follows:

$$\begin{aligned} t_{std}&\approx \alpha + \beta _{FE} \; StaffFixedEffects \; + \\&\beta _{F_{SgSc}} \;Fam_{std}^{SgSc} + \beta _{F_{SgC}} \;Fam_{std}^{SgC} +\\&\beta _{F_{CSc}} \;Fam_{std}^{CSc} . \end{aligned}$$

We incorporate staff fixed effects to block backdoor paths identified in our DAG (Section 4.1), thereby accounting for unobserved, time-invariant characteristics of individual staff members that may confound the relationship between team familiarity and procedure duration. This approach is widely used in observational research to mitigate selection bias and enhance causal identification [43]. By holding constant each staff member’s average contribution to surgery duration—such as skill, experience, and work ethic—we reduce the influence of confounding variables that could bias our estimates. In particular, staff fixed effects help address endogeneity arising from the nurse manager’s non-random assignment of nurses to procedures, which could otherwise correlate with both team familiarity and surgery duration. As such, our fixed effects regression framework supports a more credible estimation of the causal effect of team familiarity.

**Table 1:** Regression results (6mo-lookback)

	*Standardized Procedure Duration*		
	(1)	(2)	(3)
$$\beta _{F_{SgSc}}$$	−0.023$$^{***}$$	−0.031$$^{***}$$	−0.019$$^{**}$$
	(0.006)	(0.009)	(0.009)
$$\beta _{F_{SgC}}$$	−0.018$$^{***}$$	−0.016$$^{*}$$	−0.001
	(0.007)	(0.008)	(0.01)
$\beta _{F_{CSc}}$	−0.003	0.002	-0.008
	(0.007)	(0.008)	(0.009)
FE(Sg,Sc,C)	–		
FE(Sg,Sc,C,SgSc)		–	
FE(Sg,Sc,C,SgSc,SgC)			–
Observations	33374	33374	33374

*Note:* $$^{*}$$p<0.1; $$^{**}$$p<0.05; $$^{***}$$p<0.01

Given that we don’t fully know the details of how individual staff characteristics affect the nurse manager’s decision-making, we conduct sensitivity analysis by considering different potential fixed effects for integration into our model. These include individual fixed effects and pairwise fixed effects. These fixed effects serve to account for the potential consideration by the nurse manager of each team member’s individual characteristics (including the surgeon, scrub, and circulator nurses) and the dynamics between pairs, such as surgeon-scrub and surgeon-circulator pairs.

Hence, this gives rise to multiple plausible model specifications where we control for either the individual characteristics alone or a combination of individual and selected pairwise fixed effects^5^.

To ensure the robustness of our findings, we rigorously test these model specifications. They vary in their fixed effects—ranging from models with only individual fixed effects to those combining individual, surgeon-scrub, and surgeon-circulator fixed effects.

For each specification, we estimate the results across three different lookback periods (6-month, 9-month, and 12-month) to confirm the consistency of our findings. In total, 9 models (three model specifications across three lookback periods each) were tested using OLS estimation. The estimation results for all three model specifications for 6-month lookback period are presented in Table 1, while the results for the other models with 9-month and 12-month lookback periods are detailed in Appendix C. In Table 1, $\beta _{F_{SgSc}}$, $\beta _{F_{SgC}}$ and $\beta _{F_{CSc}}$ denote the coefficients for the standardized surgeon-scrub familiarity ($Fam^{SgSc}_{std}$), surgeon-circulator familiarity ($Fam^{SgC}_{std}$) and circulator-scrub familiarity ($Fam^{CSc}_{std}$) , respectively.

Across all 9 models, we estimated statistically significant negative coefficients for the effect of familiarity between surgeon and scrub nurse on procedure duration. Hence, under the DAG assumptions, we argue that there is a negative causal relationship between the level of familiarity between scrub nurses and surgeons, and the duration of surgical procedures. In other words, as the familiarity between the attending surgeon and scrub nurse increases, surgical procedures tend to be shorter. The effect sizes for the standardized surgeon-scrub familiarity ranged from -0.019 to -0.037. We also obtained negative coefficients for surgeon and circulator nurse familiarity across all 9 models, out of which 5 are statistically significant. In most of these models, the effect size for the surgeon-circulator familiarity was smaller than the effect size for the surgeon-scrub familiarity. We did not observe any statistically significant effect of the familiarity between scrub and circulator nurses on procedure duration.

The key takeaway from these various model specifications is that our results remained consistent and dependable, further reinforcing the validity of our initial observations regarding the relationship between surgeon-scrub familiarity and procedure time. We discuss the implications of these results in the following section.

**Fig. 14:**
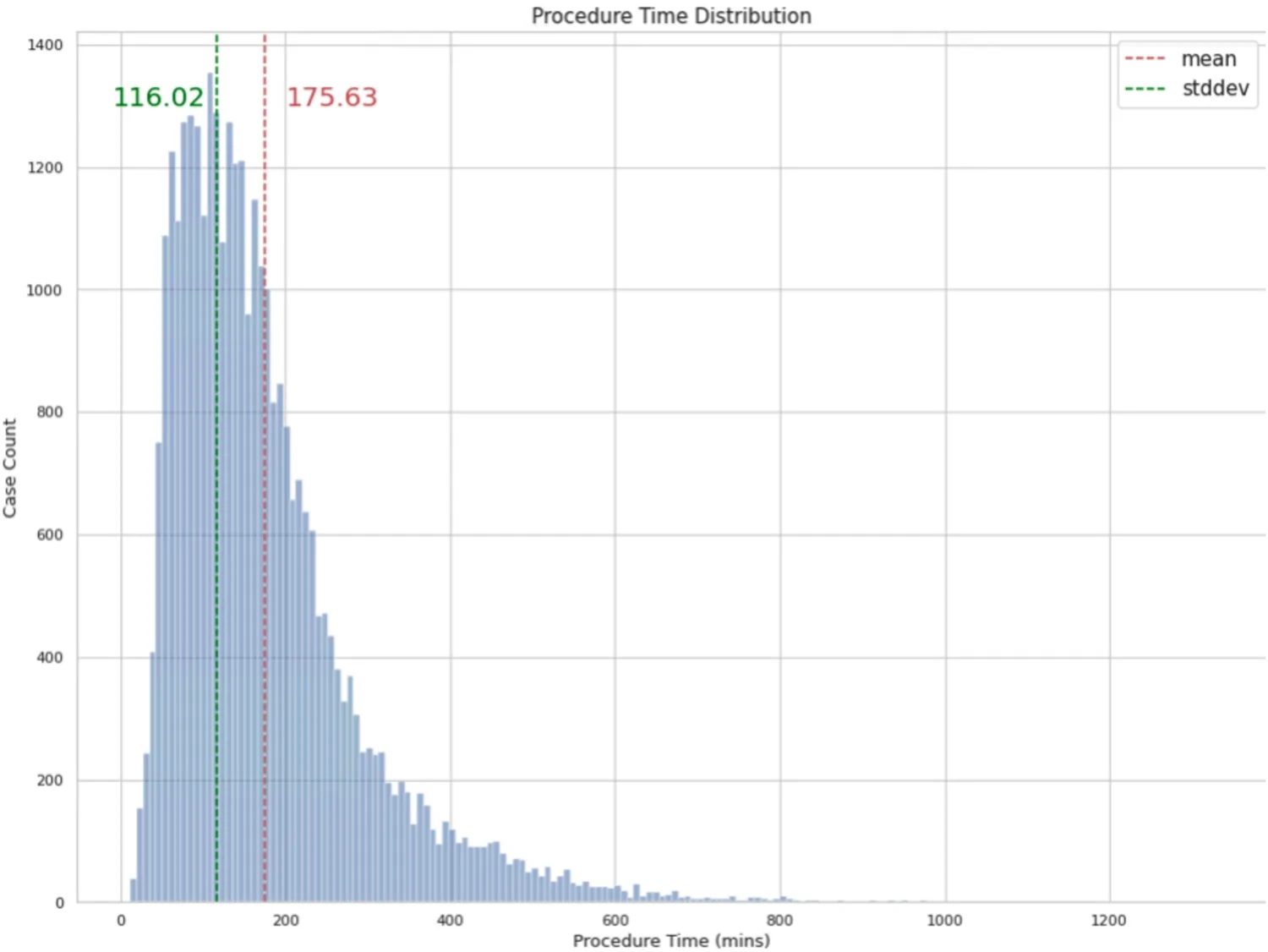
Procedure time distribution

**Table 2:** Procedure statistics

Procedure	$\mu _t$	$\sigma _t$	$\mu _{Fam^{SgSc}}$	$\sigma _{Fam^{SgSc}}$	$\mu _{Fam^{SgC}}$	$\sigma _{Fam^{SgC}}$	$\Delta _t$ (min)
Bypass Femoral Tibial	380	152	16	19	6	10	[−5.776, −2.888]
Laparotomy	258	139	1	3	2	3	[−5.282, −2.641]
Transplant Heart	557	125	14	8	14	17	[−4.75, −2.375]
Orif Humerus Fracture	236	89	8	9	13	19	[−3.382, −1.691]
AV Fistula Insertion	119	33	8	14	12	19	[−1.254, −0.627]

## Discussion

To provide a more concrete understanding of this relationship, consider a scenario where we measure team familiarity using a standard deviation scale. When we increase the familiarity score for surgeon-scrub pair by 1 standard deviation for a particular medical procedure, we observe a corresponding decrease in procedure time, with the reduction amounting to 0.019 – 0.037 standard deviations. To illustrate, let’s take the example of kidney transplant cases, where the standard deviation for procedure time was found to be 100 minutes. The standard deviation observed in the levels of familiarity in the context of kidney transplant cases is 14 for surgeon-scrub interactions. This implies that an increase in collaborative experiences by 14 cases between a surgeon and a scrub nurse is associated with a reduction in the procedure duration by 1.9-3.7 minutes for a kidney transplant case. While a 1.9 to a 3.7-minute reduction in surgery time might appear relatively small on an individual basis, it becomes significant when viewed in the context of the overall volume of cases in a hospital. In our data, the standard deviation of the procedure time across all procedures is 116.02 minutes, see Fig. 14. The standard deviation of the surgeon-scrub and surgeon-circulator familiarity across all procedures are 13.2 and 10.8, respectively. See Figs. 3 and 4 for the distribution of these quantities. Hence, increasing surgeon-scrub familiarity by 13 cases can lead to a 2 to 4 minute reduction in procedure duration. Table 2 provides other example procedure types and the associated time savings with 1 standard deviation increase in surgeon-scrub familiarity. Table 5 in Appendix D presents the same statistic for the 25 procedure types that are performed most frequently in our hospital.

We acknowledge that the observed change in procedure duration may appear modest at first glance. However, as previously discussed, our primary interest in examining procedure duration extends well beyond its direct implications for operational efficiency. Shorter procedure times in the OR have been consistently associated with meaningful improvements across several critical domains, including reduced surgical errors and adverse events, enhanced overall quality of care, decreased resource waste, and improved patient safety. Additionally, we regard procedure duration as an important proxy for team satisfaction—a well-recognized driver of broader organizational benefits.

Moreover, these findings align with similar results in the literature, such as Jones et al. (2022), which highlights the impact of operational improvements on healthcare worker satisfaction, and Witmer et al. (2022), which provides a comprehensive review of studies demonstrating similar cost and efficiency gains. These studies underscore that the effect sizes observed in our research are not only relevant but also consistent with broader trends in healthcare operations, further emphasizing the practical value of our findings in real-world hospital settings.

To gain a more comprehensive understanding, one might refer to Witmer (2022) [60], which provides an in-depth review of the time savings documented in the existing literature. What is particularly noteworthy is the parallel between the general estimates found in these studies and our results. When we distill our broad findings encompassing a wide array of procedures to individual procedures, the numbers align closely with those in the literature.

For instance, Kirksey (2022) [31] reported that for total knee arthroplasty procedures, a 10-percentage point rise in the consistency score^6^ corresponds to a 3.4 minute reduction in surgery duration. In our dataset, the standard deviation for the durations of total knee arthroplasty procedures is 30. From this, we infer that a one standard deviation increase in the familiarity between the surgeon and the scrub team can shorten the surgery time by approximately 0.57 to 1.11 minutes. This estimation is derived by multiplying the effect sizes we observed, which range from -0.019 to -0.037 when expressed in units of standard deviation, with the standard deviation itself. Although the metrics of familiarity differ between the studies, making a direct comparison challenging, the similarity in the magnitude of the values is noteworthy. Our study, focusing on the causal effects implied by a DAG model rather than mere correlations, understandably yields slightly lower values, which aligns with our expectations.

This specific range is in harmony with our estimates, reinforcing the consistency and reliability of our results. This coherence across various research efforts highlights the significant role that familiarity plays in enhancing efficiency, particularly in medical procedures.

## Limitations

Teamwork is a nuanced and unpredictable outcome of interactions between individuals, making it difficult to measure objectively. This complexity arises from human elements like motivation, communication, contentment, awareness of circumstances, and emotional intelligence [33]. Hence, there are no uniform metrics or established benchmarks for measuring teamwork. Consequently, varying studies employ distinct team metrics, posing challenges for direct comparisons between them.

There are also several limitations specific to this study. First of all, our study is based on observational data, not based on a randomized control trial, which is the golden standard of causal inference. However, our setting is not very suitable for a randomized control trial due to patient safety concerns. Moreover, the nurse assignments are not performed randomly and studies can suffer from a potential selection bias. This gives rise to a modeling approach that aims to control selection bias by understanding the data generation process. This results in multiple plausible causal model specifications in our context and an estimation range for the effect size of team familiarity on procedure duration. Additionally, our study is conducted in the US context. We acknowledge that OR staff and their roles may vary by country; and our results may not be generalizable to other countries. Finally, our study does not evaluate the impact of shorter procedure times on surgical precision or clinical outcomes.

Increased team familiarity brings undeniable benefits, but practical obstacles like dynamic schedules, staff availability, job transitions, and diverse case types present hurdles to achieving “perfect” teaming, resulting in surgical teams with low levels of familiarity. Our study focuses on procedure duration as an outcome and does not directly capture data on complication rates or quality-related endpoints. While our observational design reflects real-world conditions in the absence of incentives for speed, we cannot definitively rule out that faster procedures compromise precision. However, prior work by Jones et al. (2022) found that greater familiarity among OR team members was associated with higher intraoperative satisfaction scores, which suggests that rushed or low-quality care is unlikely to be a systematic result of team familiarity. We view this as a valuable area for future research, particularly in settings that explicitly incentivize efficiency or speed.

Increased team familiarity brings undeniable benefits, but practical obstacles like dynamic schedules, staff availability, job transitions and diverse case types present hurdles to achieving “perfect” teaming, resulting in surgical teams with low levels of familiarity.

Despite these factors, increased team familiarity holds significant improvement in team continuity remains achievable at large medical centers like ours. Despite these challenges, substantial potential exists for enhancing teaming practices within large medical centers, as demonstrated by our findings.

## Conclusions

Extensive research has established the advantages of surgical teaming and relational coordination. This has led to an increased focus on integrating structured teamwork throughout surgical care, including the intraoperative setting [11].

This study delves into the critical realm of enhancing team performance within real hospital operating rooms by closely examining the impact of team familiarity. Unlike previous research that often focused on specific surgical procedures, we include a wide range of surgical procedures conducted in a high-volume hospital within our analysis. Our primary focus was on developing a DAG model which, if holds true, identifies a causal relationship between team familiarity and surgical duration across all types of procedures. Specifically, we placed significant emphasis on exploring the effects of familiarity between the surgeon and both the scrub nurse and the circulator nurse, recognizing the pivotal roles these individuals play in surgical teams. Hence, our study is the first to examine the hospital-wide relationship between surgical team familiarity and procedure duration using a causal inference approach.

Our analysis examined various plausible model specifications, consistently revealing a statistically significant and negative coefficient associated with the level of familiarity between the surgeon and the scrub nurses. We also obtained negative coefficients for the surgeon-circulator familiarity across all 9 models, out of which 5 are statistically significant. This finding underscores the importance of team familiarity, as an increase in familiarity leads to a substantial reduction in procedure duration. Team familiarity within surgical teams can be leveraged to optimize team performance within the setting of a hospital OR and can be a consideration for OR scheduling practices. Considering the team familiarity in OR scheduling practices holds significant implications for improving the efficiency of surgical teams and, by extension, enhancing patient care within the hospital operating room.

## Figures and Tables

**Fig. 15:**
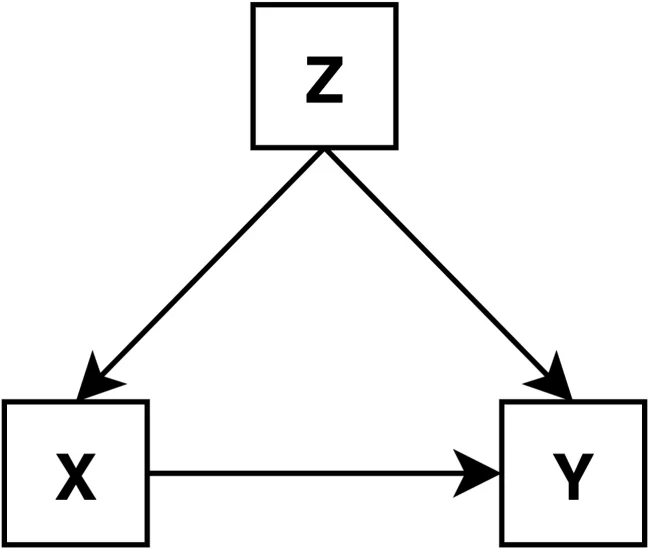
A directed acyclic graph (DAG) representing the causal structure between three variables, X, Y, and Z

## Data Availability

Data is proprietary and covered by HIPAA law. Hence, it is not available.

## Appendix A: Structural Causal Modeling (SCM) framework

Structural causal modeling (SCM) [44] is a causal inference framework that integrates structural equation modeling (SEM) [52, 64] with Rubin’s potential outcome (PO) framework [49], among others. A key feature of SCM is its ability to identify cause-and-effect relationships from observational data, mitigating the need for randomized control experiments [2, 44].

In the Structural Causal Modeling (SCM) approach, the calculation of causal effects is grounded in a series of assumptions about how the data is generated. These assumptions are graphically represented through DAGs [41] [44]. A DAG is composed of various nodes (representing variables) that are interconnected through edges (depicted as arrows). These arrows symbolize the causal links between variables, extending from the cause to the effect, indicating that causes always come before their effects.

For instance, the DAG depicted in Fig. 15 illustrates that X has a direct effect on Y (X → Y), while Z directly influences both X (Z → X) and Y (Z → Y), and also affects Y indirectly through X (Z → X → Y). It’s crucial to understand that the arrows linking the nodes (variables) in a DAG symbolize proposed causal relationships. Conversely, when two nodes lack a connecting arrow, it implies no causal relationship is assumed between those variables, reflecting strong initial causal assumptions. Hence, the absence of arrows in a DAG encodes these causal assumptions, whereas the presence of arrows indicates the potential for an effect [2, 15]. Directed acyclic graphs (DAGs) are nonparametric in nature, which implies that they do not presuppose any specific characteristics regarding the random behavior of variables, their measurement, or the nature of direct impacts, such as being linear, nonlinear, or stepwise, nor do they assume anything about the magnitude of these effects [20].

After constructing a Directed Acyclic Graph (DAG), it can be tested against observational data to verify its consistency with the data. Essentially, a DAG proposes various independencies expected to be observed in the data, assuming the DAG and the observational data accurately reflect the data-generating process. Within a DAG, two variables, such as X and Y, can be independent if there are no connecting paths (a series of nodes and arrows) between them. Additionally, variables can also exhibit conditional independence under certain conditions. These independence assumptions can be tested using the data via conditional independence tests. A DAG may require a series of modifications until consistency between the DAG and the data is achieved. Ankan et al. (2021) provide guidelines on updating DAGs to achieve DAG–data consistency.

Once the DAG is created and validated against the data, the next step is to apply either the backdoor or frontdoor criterion. Given the configuration of our DAG, the backdoor criterion is more applicable in our context and thus will be the primary focus. For those interested in exploring the frontdoor criterion, as well as additional details on the backdoor criterion, we recommend references to Pearl’s works from 2009 [44] and 2016 [45].

A crucial tool employed to assess whether we can compute a causal effect involves a simple test referred to as the *backdoor criterion*. By utilizing this criterion, we can determine the appropriate set of variables Z within the causal model depicted by a Directed Acyclic Graph (DAG) on which to condition when searching for a causal relationship between any two variables, X and Y [45]. This approach works only when Z is observable. If this is not true, the frontdoor criterion needs to be used. As all variables in the key nodes of our DAG are observable, we use only the backdoor criterion and refer the reader interested in frontdoor criterion to Pearl’s works from 2009 [44] and 2016 [45].

**Definition (The Backdoor Criterion)** *Given an ordered pair of variables (X, Y) in a directed acyclic graph G, a set of variables Z satisfies the backdoor criterion relative to (X, Y) if no node in Z is a descendant of X, and Z blocks every path between X and Y that contains an arrow into X* [45].

Following this logic, we need to condition on a set of nodes Z such that

- We block all spurious paths between X and Y.
- We leave all directed paths from X to Y unperturbed.
- We create no new spurious paths.

To find the causal effect of X on Y, we want the nodes we condition on to block any “backdoor” path from Y in which one end has an arrow into X, because such paths may make X and Y dependent, but are obviously not transmitting causal influences from X, and if we do not block them, they will confound the effect that X has on Y. We condition on backdoor paths so as to fulfill our first requirement. However, we don’t want to condition on any nodes that are descendants of X. Descendants of X would be affected by an intervention on X and might themselves affect Y; conditioning on them would block those pathways. Therefore, we don’t condition on descendants of X so as to fulfill our second requirement. Finally, to comply with the third requirement, we should refrain from conditioning on any collider^7^ that would unblock a new path between X and Y. The requirement of excluding descendants of X also protects us from conditioning on children of intermediate nodes between X and Y. Such conditioning would distort the passage of causal association between X and Y, similar to the way conditioning on their parents would [45]. For example, assume that we are interested in the causal effect of X (treatment) on Y (outcome) depicted in Fig. 15. However, there is a backdoor path X← Z → Y, which creates a noncausal pathway. Notice that Z blocks this backdoor path and satisfies the backdoor criterion.

Once we identify the set of variables Z that satisfy the backdoor criterion, we choose a statistical model to estimate the causal effect of interest by controlling for Z. This concludes a brief overview of the SCM framework. In Section 4, we employ this framework to identify the causal effect of team familiarity on procedure duration.

## Appendix B: Model testing

Our DAG model structure (Fig. 11) implies the following relationships among variables that require testing, which are identified using R package ‘dagitty’ and its web tool dagitty.net:

$$\begin{aligned}&E_C \perp F_{CS} \,|\, C \;  &   E_C \perp E_{Sc} \,|\, Sc \\&E_C \perp E_{Sc} \,|\, Sg \;  &   E_C \perp E_{Sc} \,|\, C \\&E_C \perp E_{Sg} \,|\, Sg \;  &   E_C \perp E_{Sg} \,|\, C\\&E_C \perp F_{SC} \,|\, C \;  &   E_C \perp F_{SS} \,|\, Sg \\&E_C \perp F_{SS} \,|\, C\;  &   E_{Sc} \perp E_{Sg} \,|\, Sg \\&E_{Sc} \perp E_{Sg} \,|\, Sc\;  &   E_{Sc} \perp F_{CS} \,|\, Sc \\&E_{Sc} \perp F_{SC} \,|\, Sg \;  &   E_{Sc} \perp F_{SC} \,|\, Sc \\&E_{Sc} \perp F_{SS} \,|\, Sc\;  &   E_{Sg} \perp F_{CS} \,|\, C, Sc \\&E_{Sg} \perp F_{CS} \,|\, Sg \;  &   E_{Sg} \perp F_{SC} \,|\, Sg \\&E_{Sg} \perp F_{SS} \,|\, Sg \;  &   F_{CS} \perp F_{SC} \,|\, C\\&F_{CS} \perp F_{SS} \,|\, Sc \;  &   F_{SC} \perp F_{SS} \,|\, Sg, \\&E_C \perp F_{SS} \,|\, Sc \;  &   E_{Sc} \perp F_{SC} \,|\, C \\&E_{Sg} \perp F_{SC} \,|\, C \;  &   E_{Sg} \perp F_{SC} \,|\, C \\&E_{Sg} \perp F_{SS} \,|\, Sc \;  &   F_{SC} \perp F_{SS} \,|\, Sc, \\&F_{SC} \perp F_{SS} \,|\, C, \; \end{aligned}$$

where

$$\begin{aligned}&E_C: \text {Circulator Service Line Experience}^* ,\\&E_{Sc}: \text {Scrub Service Line Experience}^*,\\&E_{Sg}: \text {Surgeon Experience}^*,\\&F_{SC}: \text {Surgeon-Circulator Familiarity}^*,\\&F_{SS}: \text {Surgeon-Scrub Familiarity} ^*,\\&F_{CS}: \text {Circulator-Scrub Familiarity} ^*, \\&C: \text {Circulator(fixed effect)},\\&Sc: \text {Scrub(fixed effect)}, \\&Sg: \text {Surgeon(fixed effect)}, \end{aligned}$$

and $^*$ denote standardized metrics.

## Appendix C: Additional model specifications

As discussed in Section 4.3, we don’t fully know the influence of staff-specific factors on the nurse manager’s decision-making process. Thus, we are exploring various potential fixed effects to incorporate into our model for sensitivity analysis. These include individual fixed effects and pairwise fixed effects. These fixed effects are considered to capture the potential influence of individual traits of team members (such as the surgeon, scrub, and circulator nurses) and the dynamics between pairs, specifically surgeon-scrub and surgeon-circulator, on the decision-making process. In addition, after modifying the structure of the Directed Acyclic Graph (DAG) due to a suspected violation of conditional independence, as discussed in Section 4.2, we have adjusted our model specifications to include service line experience. To ensure the robustness of our results, we have conducted estimations for each model specification over three different lookback periods: 6 months, 9 months, and 12 months. This approach resulted in the testing of a total of 9 models, encompassing six different model specifications over the three specified lookback periods, using Ordinary Least Squares (OLS) estimation. The estimation outcomes for all three models with 6-month lookback period are displayed in Table 1 in Section 4.3. The findings for models with the 9 and 12-month lookback periods are presented in this section and descriptions of the symbols used in the regression results are presented below. Note that we do not report the coefficients for the fixed effects as there are thousands of such coefficients.

$$\begin{aligned}&\beta _{F_{SS}}: \text {coefficient of standardized surgeon-scrub familiarity} (Fam^{SS}_{std}),\\&\beta _{F_{SC}}: \!\text {coefficient of standardized surgeon-circulator familiarity} (Fam^{SC}_{std}),\\&\beta _{F_{CS}}: \text {coefficient of standardized circulator-scrub familiarity} (Fam^{CS}_{std}).\\ \end{aligned}$$

### C.1 Models with 9-mo lookback period

The results for the models using 9-month lookback period are presented in Table 3.

**Table 3:** Regression Results (9mo-lookback)

	*Standardized Procedure Duration*		
	(1)	(2)	(3)
$$\beta _{F_{SS}}$$	−0.022$$^{***}$$	−0.027$$^{***}$$	−0.021$$^{**}$$
	(0.007)	(0.008)	(0.001)
$$\beta _{F_{SC}}$$	−0.019$$^{***}$$	−0.013$$^{*}$$	−0.003
	(0.007)	(0.008)	(0.01)
$\beta _{F_{CS}}$	0.003	0.005	-0.007
	(0.007)	(0.008)	(0.009)
FE(Sg,Sc,C)	–		
FE(Sg,Sc,C,SS)		–	
FE(Sg,Sc,C,SS,SC)			–
Observations	34757	34757	34757

*Note:* $$^{*}$$p<0.1; $$^{**}$$p<0.05; $$^{***}$$p<0.01

### C.2 Models with 12-mo lookback period

The results for the models using 12-month lookback period are presented in Table 4.

**Table 4:** Regression results (12mo-lookback)

	*Standardized Procedure Duration*		
	(1)	(2)	(3)
$$\beta _{F_{SS}}$$	−0.025$$^{***}$$	−0.037$$^{***}$$	−0.033$$^{***}$$
	(0.007)	(0.009)	(0.01)
$$\beta _{F_{SC}}$$	−0.015$$^{***}$$	−0.01	−0.005
	(0.007)	(0.008)	(0.011)
$\beta _{F_{CS}}$	0.000	0.003	−0.008
	(0.007)	(0.008)	(0.009)
FE(Sg,Sc,C)	–		
FE(Sg,Sc,C,SS)		–	
FE(Sg,Sc,C,SS,SC)			–
Observations	33374	34757	35028

*Note:* $$^{*}$$p<0.1; $$^{**}$$p<0.05; $$^{***}$$p<0.01

## Appendix D: Procedure statistics

Table 5 provides team familiarity and procedure duration statistics for the 25 most frequently performed procedures in our institution.

**Table 5:** Procedure statistics

Procedure	$\mu _t$	$\sigma _t$	$\mu _{Fam^{SS}}$	$\sigma _{Fam^{SS}}$	$\mu _{Fam^{SC}}$	$\sigma _{Fam^{SC}}$	$\Delta _t$ (min)	N
1	99	41	22	19	13	15	[−0.8, −1.5]	813
2	62	21	8	12	4	6	[−0.4, −0.8]	669
3	178	28	15	17	12	14	[−0.5, −1]	568
4	194	91	1	2	1	1	[−1.7, −3.4]	557
5	451	77	14	8	15	18	[−1.5, −2.9]	520
6	206	103	2	6	1	3	[−2, −3.8]	502
7	99	60	5	9	4	7	[−1.1, −2.2]	456
8	158	52	4	11	2	5	[−1, −1.9]	414
9	199	30	30	24	23	19	[−0.6, −1.1]	409
10	175	37	11	10	10	9	[−0.7, −1.4]	332
11	246	53	3	3	1	2	[−1, −2]	315
12	185	30	34	24	20	17	[−0.6, −1.1]	313
13	138	27	16	22	6	8	[−0.5, −1]	271
14	220	101	2	6	1	3	[−1.9, −3.7]	250
15	165	80	3	4	3	4	[−1.5, −3]	250
16	143	43	8	9	10	19	[−0.8, −1.6]	241
17	327	120	9	8	7	6	[−2.3, −4.4]	238
18	237	51	6	10	3	6	[−1, −1.9]	234
19	123	38	2	4	2	4	[−0.7, −1.4]	233
20	62	24	8	12	4	6	[−0.5, −0.9]	232
21	119	33	12	19	8	14	[−0.6, −1.2]	228
22	236	73	23	24	15	17	[−1.4, −2.7]	224
23	239	72	23	23	15	17	[−1.4, −2.7]	223
24	76	32	8	12	3	4	[−0.6, −1.2]	217
25	121	53	6	12	6	12	[−1, −2]	216

## Footnotes

1 A sterile field is an area created by placing sterile surgical drapes around the patient’s surgical site and on the stand that will hold sterile instruments and other items needed during surgery.

2 We also perform robustness checks for 9 and 12 months.

3 Procedure duration refers to the time that a given surgical procedure takes per our definition in Section 3.4.

4 Preference cards are catalogs of the specific tools, supplies, and room setup that a surgeon prefers to use for a particular type of surgical case.

5 We do not consider a triplet fixed effect as it is unlikely that the nurse manager considers the characteristics of a triplet altogether in the assignment process.

6 This score is calculated as a percentage, based on a weighted graph of all team members who have collaborated with the surgeon during the 90 days leading up to the specific surgery.
